# Drug-induced orthostatic hypotension: A systematic review and meta-analysis of randomised controlled trials

**DOI:** 10.1371/journal.pmed.1003821

**Published:** 2021-11-09

**Authors:** Cini Bhanu, Danielle Nimmons, Irene Petersen, Mine Orlu, Daniel Davis, Hajra Hussain, Sanuri Magammanage, Kate Walters

**Affiliations:** 1 Research Department of Primary Care and Population Health, University College London, United Kingdom; 2 UCL School of Pharmacy, University College London, United Kingdom; 3 MRC Unit for Lifelong Health & Ageing, University College London, United Kingdom; Columbia University, UNITED STATES

## Abstract

**Background:**

Drug-induced orthostatic hypotension (OH) is common, and its resulting cerebral hypoperfusion is linked to adverse outcomes including falls, strokes, cognitive impairment, and increased mortality. The extent to which specific medications are associated with OH remains unclear.

**Methods and findings:**

We conducted a systematic review and meta-analysis to evaluate the extent to which specific drug groups are associated with OH. EMBASE, MEDLINE, and Web of Science databases were searched from inception through 23 November 2020. Placebo-controlled randomised controlled trials (RCTs) on any drug reporting on OH as an adverse effect in adults (≥18 years) were eligible. Three authors extracted data on the drug, OH, dose, participant characteristics, and study setting. The revised Cochrane risk-of-bias tool for randomised trials (RoB 2) was used to appraise evidence. Summary odds ratios (ORs) were estimated for OH using fixed effects Mantel–Haenszel statistics. We conducted subgroup analysis on validity of OH measurement, drug dose, risk of bias, age, and comorbidity. The Grading of Recommendations Assessment, Development, and Evaluation (GRADE) tool was used to summarise the certainty of evidence. Of 36,940 citations, 69 eligible RCTs were included in the meta-analysis comprising 27,079 participants. Compared with placebo, beta-blockers and tricyclic antidepressants were associated with increased odds of OH (OR 7.76 [95% CI 2.51, 24.03]; OR 6.30 [95% CI 2.86, 13.91]). Alpha-blockers, antipsychotics, and SGLT-2 inhibitors were associated with up to 2-fold increased odds of OH, compared to placebo. There was no statistically significant difference in odds of OH with vasodilators (CCBs, ACE inhibitors/ARBs, SSRIs), compared to placebo. Limitations of this study are as follows: data limited to placebo-controlled studies, (excluding head-to-head trials), many RCTs excluded older participants; therefore results may be amplified in older patients in the clinical setting. The study protocol is publicly available on PROSPERO (CRD42020168697).

**Conclusions:**

Medications prescribed for common conditions (including depression, diabetes, and lower urinary tract symptoms) were associated with significantly increased odds of OH. Drugs causing sympathetic inhibition were associated with significantly increased odds of OH, while most vasodilators were associated with small nonsignificant differences in odds of OH, compared to placebo. Drugs targeting multiple parts of the orthostatic blood pressure (BP) reflex pathway (e.g. sympathetic inhibition, vasodilation, cardio-inhibitory effects) may carry cumulative risk, suggesting that individuals with polypharmacy could benefit from postural BP monitoring.

## Abstract 2

Cini Bhanu and colleagues evaluate the extent to which different drug groups are associated with orthostatic hypertension in this systematic review and meta-analysis.

## Author summary

**Why was this study done?:**

- Orthostatic hypotension (OH) is a common side effect of drugs. It causes a reduction in blood pressure (BP) on standing, which results in reduced cerebral blood flow that is linked to falls, strokes, cognitive impairment, and increased mortality.
- Over 250 medications are associated with OH.
- However, there is conflicting evidence on the extent to which different drug groups are associated with OH as a side effect.
- To our knowledge, there are no systematic reviews providing an overview of which drugs are associated with OH.

**What did the researchers do and find?:**

- We conducted a systematic review and meta-analysis of randomised controlled trials (RCTs) comparing a drug to placebo, including 69 trials comprising 27,079 participants, to address the extent to which specific medications are associated with OH.
- We found that drugs primarily inhibiting sympathetic activity were associated with significantly increased odds of OH, compared to placebo (beta-blockers, TCAs, antipsychotics, alpha-blockers).
- Drugs primarily causing vasodilation (CCBs, ACE inhibitors/ARBs, SSRIs, SGLT-2 inhibitors) were associated with smaller differences that were not statistically significant, compared to placebo.

**What do these findings mean?:**

- Drugs prescribed widely for common conditions including lower urinary tract symptoms, mental health conditions, pain, and insomnia are associated with significantly increased risk of OH; alternative prescribing, shorter treatment, and postural BP checks should be considered to manage this risk.
- The relationship between antihypertensives and antidiabetic drugs and OH is more complex.
- Drugs targeting multiple parts of the BP reflex pathway causing OH may carry cumulative risk, suggesting that individuals with polypharmacy could benefit from routine postural BP monitoring.

## Introduction

Orthostatic hypotension (OH), defined as a reduction in systolic blood pressure (BP) of ≥20 mm Hg or diastolic BP of ≥10 mm Hg within 3 minutes of assuming an erect posture [1], is estimated to affect 30% to 70% of older adults [2] and is commonly associated with use of medications [3]. OH and its resulting cerebral hypoperfusion is linked to falls [4.^5^], fractures [5], ischaemic events [2,5], cognitive impairment [6], and mortality [5]. Over 250 medications are associated with OH [3], and the incidence of OH increases with advancing age [6]. As polypharmacy rises worldwide in the ageing population [7], drug-induced OH is of greater concern.

The physiological transition from a supine to upright position involves redistribution of intravascular volume causing a transient reduction in venous return, a decrease in stroke volume, cardiac output, and BP. In a normal response, activation of BP-regulating reflexes leads to stimulation of the sympathetic system, increasing heart rate, venous return, cardiac contractility, and vascular tone, eventually restoring BP within seconds [8]. Drug-induced OH can impair mechanisms in this process [9].

A recent narrative review identified a range of cardiovascular and psychoactive drugs causing OH [10]. A further narrative review found that evidence for OH induced by antihypertensives was weak [11], and a recent meta-analysis of randomised controlled trials (RCTs) found that intensive BP-lowering treatment reduces OH risk [12]. However, cross-sectional studies have found an increased risk of OH with specific antihypertensive classes [13,14]; retrospective cohort studies have identified antihypertensives, antidepressants, and alpha-blockers [4,15] as strongly associated with OH, with a cumulative risk when combined [4]. Such observational studies can be subject to unmeasured confounding, including confounding by indication, compared to RCTs [16]. To address the extent to which specific medications might be associated with OH, we undertook a systematic review and meta-analysis of placebo-controlled RCTs reporting which drugs are associated with adverse OH in adults (≥18 years).

## Methods

### Data sources and searches

The search strategies (S1 Text) were developed without language restrictions, and the databases EMBASE, MEDLINE, and Web of Science were searched from inception to November 23, 2020. A search strategy for general drug terms, individual drug names (identified by the international nonproprietary name (INN) in the British National Formulary (BNF) and US National Library of Medicines) were used, and drugs with name changes were accounted for [17]. To facilitate the search strategy, a macro was developed to automate searches (Pulover’s macro creator version 5.2.8). Reference lists of eligible reports were reviewed, and authors were contacted to supplement incomplete papers. This study is reported as per the Preferred Reporting Items for Systematic Reviews and Meta-Analyses (PRISMA) guideline (S1 Checklist). The study protocol is publicly available on PROSPERO (CRD42020168697).

### Study selection

RCTs comparing any drug with placebo, reporting OH as an adverse effect or outcome in adults (≥18 years), were included. Studies in pregnancy and anaesthetic or surgical contexts were excluded. Studies on the drug treatment of OH (such as midodrine, fludrocortisone, droxidopa, erythropoietin, pyridostigmine) and investigational or withdrawn drugs were excluded.

### Data extraction and quality assessment

Using a standardised form, 1 reviewer (CB) screened all titles, abstracts, and full-text articles reporting potentially eligible studies. A second reviewer (DN) screened 10% of titles and abstracts and 3 reviewers (DN, HH, and SM) screened 10% of all full-text articles. Disagreements were resolved by discussion with an adjudicator (KW) when necessary. Online systematic review software (Rayyan, QCRI) was used to facilitate literature screening.

CB, HH, and SM independently extracted data from each article. Data were extracted on study characteristics, drug, dose, participant characteristics (age, sex, comorbidities), study setting, pharmaceutical sponsorship, and reporting of OH. Reporting of prevalent OH while taking the medication or placebo was grouped into 6 categories following expert consensus: “measured and validated” (a documented postural BP examination performed using a threshold of ≥20 mm Hg systolic/≥10 mm Hg diastolic reduction); “measured” (a documented postural BP examination performed without a specified threshold); “BP examined” (a documented BP examination only—but implied as postural, since the study reports OH); “physical examination”; “vital signs examination”; “symptom report” or “unclear.” Where possible, authors were contacted to obtain further information on reporting of OH. Reports of OH induced through orthostatic stress (e.g., tilt table testing) were excluded. We included RCTs comparing a drug to placebo, which reported OH according to the top 3 categories we regarded as acceptable (“measured and validated”; “measured”; “BP examined”).

Using the revised Cochrane risk-of-bias tool for randomised trials (RoB 2) [18,19], CB assessed all articles for risk of bias, and 3 reviewers (DN, HH, and SM) independently assessed 10% of articles. Studies were assigned an overall score: “low,” “some concerns,” or “high.”

The Cohen’s κ statistic addressed inter-rater agreement regarding eligibility. For duplicate studies or pooled analyses, we included the report with the most complete data. All RCTs comparing a drug versus placebo with acceptable reporting of OH were grouped according to drug class. Where at least 3 studies were available, these groups were then pooled.

### Data synthesis and analysis

Summary odds ratios (ORs) were estimated for OH (whether a patient had OH or not while taking the medication or placebo) as a dichotomous outcome using fixed effects Mantel–Haenszel statistics. Zero total event trials were included in the meta-analysis since this can move the pooled estimate of treatment effect closer to nil, decrease its confidence interval, and decrease between-study heterogeneity [20]. Heterogeneity was assessed using the I^2^ statistic with an I^2^ >30% representing substantial heterogeneity [21].

Subgroup analyses were conducted if there were 3 or more studies available in a given subgroup: (1) trials reporting “measured and validated” OH outcomes only; (2) low risk of bias (excluding studies with an overall score of “high”); (3) drug dose (low versus high); (4) older patients ≥65 years; and (5) populations at greater risk of OH (this included people with cardiovascular conditions, diabetes, and older people at high risk of falls). Review Manager software version 5.4 was used (Cochrane).

The Grading of Recommendations Assessment, Development, and Evaluation (GRADE) tool [22] was used to summarise the certainty of evidence. When there were at least 5 studies for meta-analysis, publication bias was assessed by visual assessment of funnel plot asymmetry.

## Results

**Fig 1:**
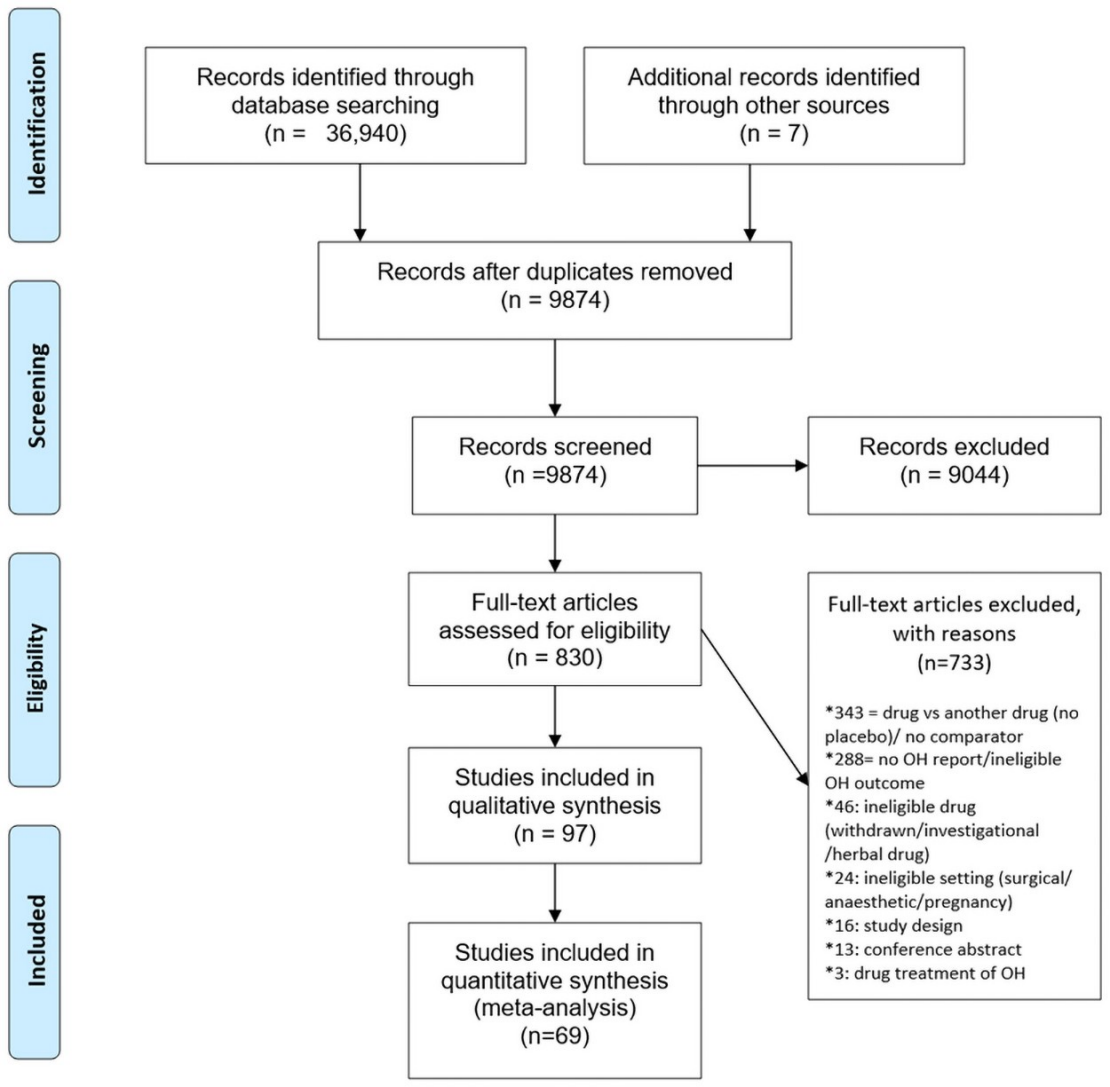
Diagram of study selection process for the systematic review and meta-analysis. OH, orthostatic hypotension.

In total, 36,940 citations were identified by the search, 9,874 citations after duplicates were removed, and 830 potentially eligible articles were retrieved in full text (Fig 1).

Overall, 69 RCTs (comprising 27,079 patients) conducted between 1970 and 2019 comparing 9 drug groups to placebo with acceptable reporting of OH were eligible for meta-analysis. Characteristics and references to studies are detailed in S1 Table.

There was substantial agreement between reviewers at the title and abstract stage (κ = 0.88) and full-text review stage (κ = 0.87). Of the 36 authors contacted for clarification of eligibility criteria and additional data, 7 responded.

40/69 studies had an overall “low” risk of bias score, and 29/69 studies scored either “some concerns” or “high” (S1 Fig). Across the 5 domains, there was substantial agreement between reviewers (κ = 0.89).

The drug classes are presented according to the key pharmacological mechanism underlying OH: vasodilators and sympathetic inhibitors.

### Vasodilators

#### Calcium channel blockers (CCBs)

**Fig 2:**
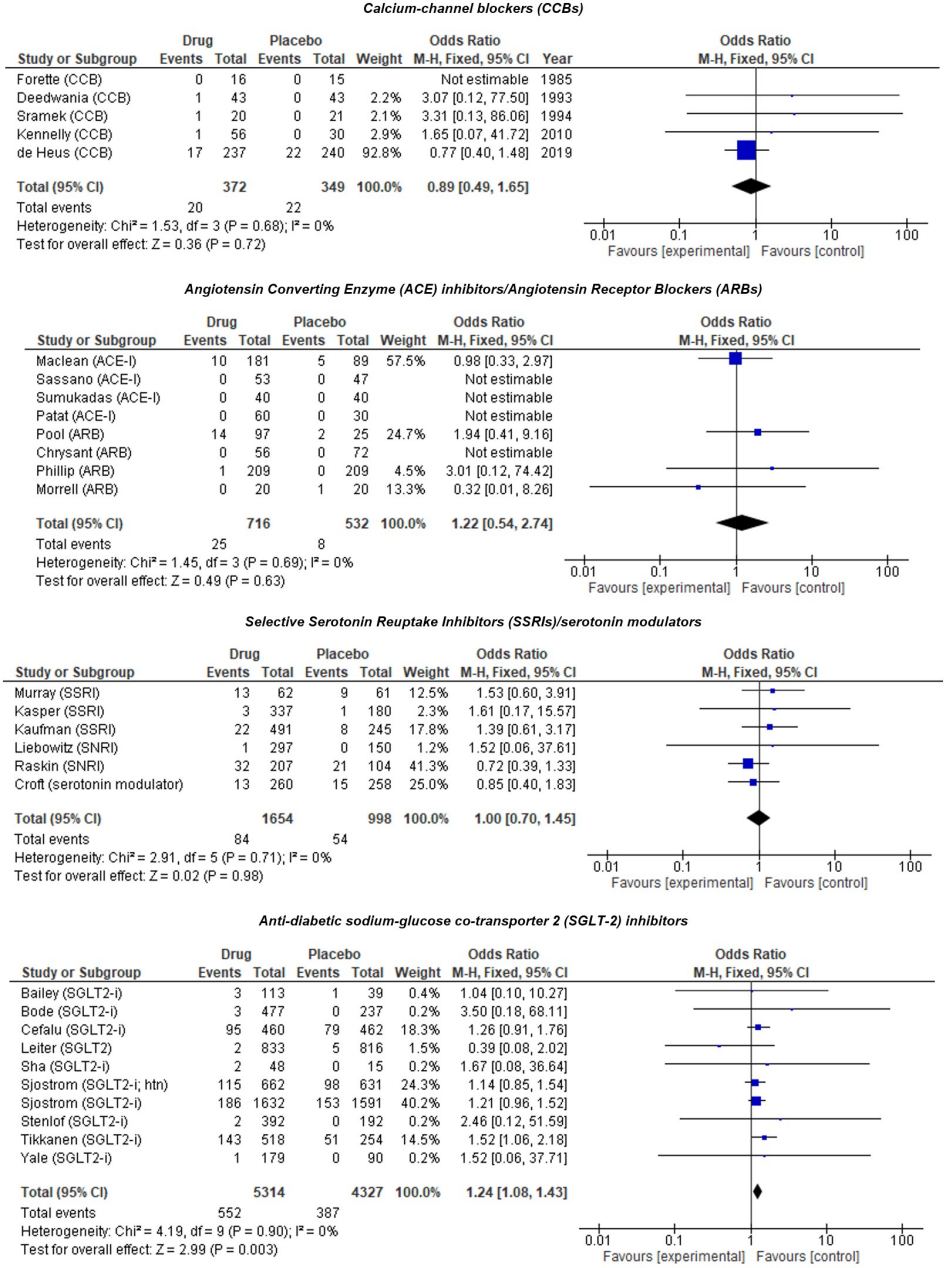
Meta-analysis results for vasodilators vs placebo. ACE, angiotensin-converting enzyme; ARB, angiotension receptor blocker; CCB, calcium channel blocker; SGLT-2, sodium–glucose cotransporter-2; SSRI, selective serotonin reuptake inhibitor.

There were 5 eligible RCTs including 721 patients comparing a CCB to placebo. The mean age among patients was 62.6 years, and 44.0% were female. The largest trial included older patients (mean age 72.4 years) (S1 Table). CCB use was associated with 11% lower odds of OH compared to placebo; however, this was nonsignificant, and a more substantial decrease or increase in odds is also possible (OR 0.89 [95% CI 0.49, 1.65]) (Fig 2). Subgroup analyses on trials with “measured and validated” outcomes only, at low risk of bias, in older patients and patients at greater risk of OH due to cardiovascular conditions showed similar results (S2 Fig).

#### Angiotensin-converting enzyme (ACE) inhibitors and angiotensin receptor blockers (ARBs)

There were 8 eligible trials including 1,248 patients comparing an ACE inhibitor or ARB to placebo. The mean age among patients was 58.3 years, and 44.7% were female (S1 Table). ACE inhibitors or ARBs were associated with 22% increased odds of OH compared to placebo; however, this was nonsignificant, and a more substantial increase or decrease in odds is also possible (OR 1.22 [95% CI 0.54, 2.74]) (Fig 2). Subgroup analysis on trials reporting “measured and validated” OH outcomes, low versus high dose, and populations at greater risk of OH (hypertension; pulmonary hypertension; older patients with falls risk) showed similar results (S2 Fig).

#### Selective serotonin reuptake inhibitors (SSRIs) and serotonin modulators

There were 6 eligible trials including 2,333 patients comparing an SSRI or serotonin modulator to placebo. The mean age among patients was 57.7 years, 49.7% were female, and the majority were treated for depression (S1 Table). SSRIs and serotonin modulators were not associated with increased odds of OH compared to placebo. However, a substantial decrease or increase in odds is possible (OR 1.00 [95% CI 0.70, 1.45]) (Fig 2). Subgroup analysis on trials with “measured and validated” OH outcomes, low versus high dose, and in older patients showed similar results (S2 Fig).

#### Sodium–glucose cotransporter-2 (SGLT-2) inhibitors

There were 10 eligible studies including 22 trials (some studies were pooled analyses including unpublished pharmaceutical data where the original study could not be located) with 9,641 patients, comparing an SGLT-2 inhibitor with placebo. The mean age of patients was 56.1 years, 34.5% were female, and all trials (excluding 1 with healthy volunteers) were in people with type 2 diabetes mellitus (T2DM) and studied high-dose SGLT-2 inhibitors (S1 Table). SGLT-2 inhibitor use was associated with increased odds of OH compared to placebo (OR 1.24 [95% CI 1.08, 1.43]) (Fig 2). Subgroup analyses in trials at low risk of bias and in patients at higher risk of OH (T2DM; T2DM and cardiovascular conditions) showed similar results (S2 Fig).

### Sympathetic inhibitors

#### Alpha-adrenoreceptor blockers

**Fig 3:**
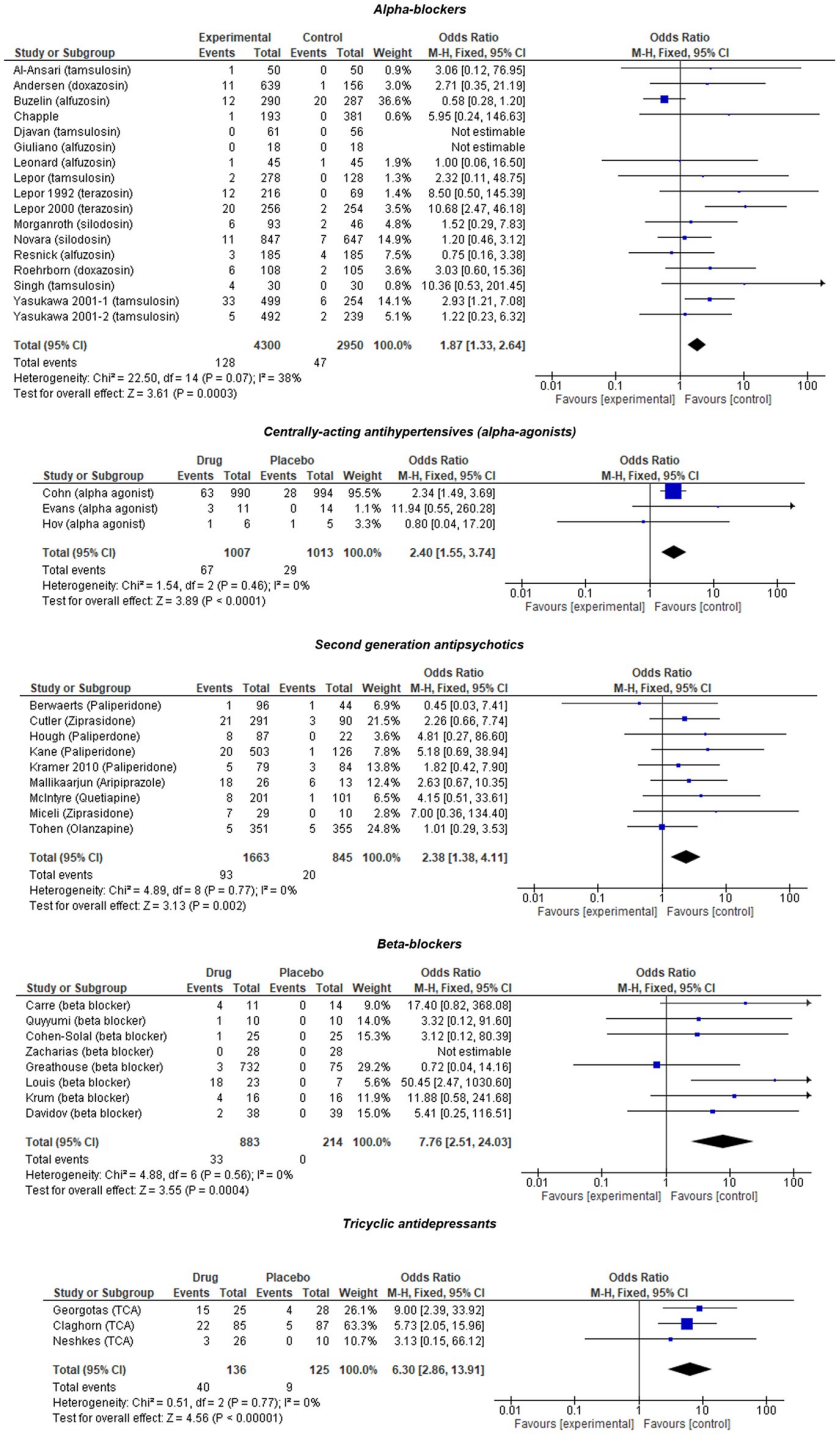
Meta-analysis results for sympathetic inhibitors vs placebo. TCA, tricyclic antidepressant.

There were 17 studies with 18 eligible trials including 7,250 patients comparing an alpha-adrenoreceptor blocker to placebo. The mean age of patients was 55.9 years, and 92.3% were males. The majority were treated for benign prostatic hypertrophy (BPH) or lower urinary tract symptoms (LUTS) at the maximum recommended dose (S1 Table). Alpha-adrenoreceptor blockers were associated with higher odds of OH compared to placebo (OR 1.87 [95% CI 1.33, 2.64]) (Fig 3). Subgroup analysis in trials with “measured and validated” OH outcomes and in populations with urological conditions (excluding healthy volunteers) showed similar results (S2 Fig).

#### Centrally acting antihypertensives

There were 3 eligible trials including 2,020 patients comparing a centrally acting antihypertensive to placebo. The mean age among patients was 71.3 years, 40.1% were female, and patients were treated for either heart failure, cor pulmonale, or acute delirium (S1 Table). Centrally acting antihypertensives were associated with increased odds of OH compared to placebo (OR 2.40 [95% CI 1.55, 3.74]) (Fig 3). There were too few studies to perform further subgroup analysis.

#### Second-generation antipsychotics

There were 9 eligible trials including 2,508 patients comparing a second-generation antipsychotic to placebo. The mean age among patients was 39.4 years, and 35.8% were female. Most trials studied oral antipsychotics for schizophrenia or psychosis (S1 Table). Second-generation antipsychotics were associated with higher odds of OH compared to placebo (OR 2.38 [95% CI 1.38, 4.11]) (Fig 3). Subgroup analysis on trials with low dose treatment (excluding high dose) and in patients with mental health conditions (excluding healthy volunteers) showed similar results. Subgroup analysis on trials with “measured and validated” OH outcomes showed no difference in odds of OH between antipsychotics and placebo (OR 1.69 [95% CI 0.90 to 3.18]) (S2 Fig).

#### Beta-blockers

There were 8 eligible trials including 1,097 patients comparing a beta-blocker to placebo. The mean age among patients was 56.1 years, and 21.0% were female (S1 Table). Beta-blockers were associated with higher odds of OH compared to placebo (OR 7.76 [95% CI 2.51, 24.03]) (Fig 3). Subgroup analysis on trials with “measured and validated” OH outcomes, low dose (excluding high dose), and in patients at greater risk of OH (hypertension; angina; heart failure) showed similar results (S2 Fig).

#### Tricyclic antidepressants (TCAs)

There were 3 eligible trials including 261 patients comparing a TCA to placebo. The mean age among patients was 53.7 years, 47.7% were female, and all trials studied major depressive disorder (S1 Table). TCAs were associated with higher odds of OH compared to placebo (OR 6.30 [95% CI 2.86, 13.91]) (Fig 3). There were too few studies to perform further subgroup analysis.

Other drug groups identified in the eligible RCTs with too few studies to perform meta-analysis included PDE-5 inhibitors; anti-diabetic glucagon-like peptide-1 (GLP-1) agonists; analgesics; first-generation antipsychotics; diuretics; alpha-2 adrenergic agonists; anti-emetics; anti-anginals; and immunomodulators. Parkinson disease (PD) drugs are reported elsewhere due to the complexities of analysing RCTs with patients on multiple PD medications with an increased risk of OH at baseline (through independent mechanisms); this requires in-depth analysis that is beyond the scope of this study.

The GRADE judgements are outlined in Tables A and B in S2 Table. The certainty of evidence varied. It was low to moderate for trials in the vasodilator group and predominantly moderate to high in the sympathetic inhibitors group.

## Discussion

### Main findings

We have conducted a systematic review and meta-analysis of 69 placebo-controlled RCTs to investigate the extent to which specific medications are associated with OH in adults. Compared with placebo, beta-blockers and TCAs were associated with a 6- to 7-fold increased odds of OH. Alpha-blockers, second-generation antipsychotics, centrally acting antihypertensives, and SGLT-2 inhibitors were associated with up to a 2-fold increased risk of OH, compared to placebo. There was no difference in odds of OH with CCBs, ACE inhibitors/ARBs, and SSRIs compared to placebo. These findings are based on varied certainty of evidence, which ranged from low to high. Our study has characterised a range of commonly prescribed drug classes according to OH risk to guide selective prescribing and monitoring of postural BP in practice.

Reports on antihypertensives and risk of OH in the literature are conflicted. There have been reports of OH with ACE inhibitors [12] and diuretics [14,9] that were not replicated in our study. It is possible that risk of OH was attenuated in our study due to the selective younger, fitter RCT population. Antihypertensives also induce OH through their therapeutic effect; such drugs often require much higher doses to observe adverse effects [23]. However, many studies suggest a protective postural effect associated with ACE inhibitors [9,24]. Juraschek and colleagues recently found that intensive BP-lowering treatment does not increase risk of OH [12]. It is known that hypertension itself increases risk of OH [13], so it is likely the complex interaction between baseline risk of OH in well-controlled hypertensive patients and antihypertensive drug effects complicates the true picture. Current consensus suggests that optimal control of BP (even among older adults) should be prioritised over potential risk of OH [12,25].

Beta-blockers induce OH through sympathetic inhibition decreasing heart rate and contractility, alongside combined independent vasodilatory effects [10,26]. Previous observational studies with strict measurement of postural BP have reported that beta-blockers are strongly associated with OH, independent of comorbidities, consistent with our results [13,27]. Current consensus states that beta-blockers should not be prescribed in preference for hypertension, due to both its potential to cause harm and lack of efficacy relative to other antihypertensives [27].

Among the drugs identified, alpha-blockers were associated with least risk. This is likely related to the majority of alpha-blockers in our study being uroselective (such as tamsulosin and alfuzosin), which have fewer cardiac effects [26]. Nevertheless, alpha-blockers almost doubled the odds of OH compared to placebo and are widely prescribed for prostatic hypertrophy to treat LUTS [28]. Dizziness and OH associated with alpha-blockers is a particular problem among older patients, and patients frequently discontinue alpha-blocker treatment in clinical practice [28]. The use of 5-alpha reductase inhibitors (5-ARIs) could reduce the need of alpha-blockers for LUTS [29]. Discontinuation of alpha-blocker treatment after 6 months in patients receiving combination therapy has been recommended and has been shown to have no significant effect on LUTS [28].

TCAs similarly exert their effects on postural BP through combined sympathetic inhibition and reduced vascular resistance [26]. Clinical guidelines support prescription of SSRIs in preference to TCAs for depression due to fewer adverse effects [10,30]. However, TCAs are still the second most prescribed antidepressant in older people (likely related to low-dose off-label use for pain and insomnia) [31]. While our study focused on TCAs at higher doses for depression and identified a 6-fold increase in odds of OH compared to placebo, it is likely that TCAs at lower doses also cause harm, especially in older adults at higher risk of OH due to a decrease in baroreflex sensitivity [32].

Interestingly, SGLT-2 inhibitors were the only drugs among vasodilators associated with significantly higher odds of OH. Their cardiac effects are exerted through diuresis and independent cardio-inhibitory effects (that are less well understood), alongside vasodilation [33]. SGLT-2 inhibitors are now considered preferential as second-line treatment for T2DM [34]. T2DM itself can increase the risk of OH, as a manifestation of autonomic neuropathy. However, improved glucose control and other positive effects on body weight related to SGLT-2 therapy can reduce the risk of neuropathic complications [34]. Therefore, the relationship between SGLT-2 use and OH in patients with T2DM is complex, similar to antihypertensives.

The drugs associated with highest odds of OH in our study (alpha-blockers, alpha-agonists, antipsychotics, beta-blockers, and TCAs) all share a common key mechanism of sympathetic inhibition causing cardioinhibitory effects [10,26]. Among these, beta-blockers and TCAs demonstrate the strongest association, with 6 to 7 times increased odds of OH compared to placebo. Both these groups induce OH through combined mechanisms of sympathetic inhibition and vasodilatory effects suggesting that OH risk rises with cumulative drug targets. This suggests that coprescription of drugs with the potential to cause OH may also result in cumulative harm, in keeping with a growing body of evidence [3,35].

### Study strengths and limitations

To our knowledge, this is the first systematic review providing an overview of which drugs are associated with OH. Strengths of this review included (1) a comprehensive literature search, enabling an overview of all prescription drugs associated with OH, the first of its kind; (2) analysis by drug mechanism relevant to clinical practice; (3) predominantly moderate to high-quality RCT evidence; and (4) detailed characterisation of RCTs and subgroup analyses that addressed differences in reporting of OH, risk of bias, dose, and patient characteristics.

This review has several limitations. The primary aim of this study was to provide an overview of all drug groups and their relative association with OH. Due to the extensive nature of this question, we limited studies to drug versus placebo RCTs reporting acceptable OH outcomes. We therefore excluded head-to-head drug comparisons, which are likely to be newer RCTs comparing drugs to existing treatment. We have reported the prevalence of adverse OH during medication or placebo use in trials; however, due to limited data in the included studies, we cannot be sure if all participants were established to have a normal postural BP prior to treatment. While we included a large number of RCTs overall in this study, for some drug groups, the meta-analysis results have been driven by only a few trials that carried greatest weight. We also grouped drugs by classes and acknowledge that not all individual drugs within a class will be similarly associated with OH. Future research should examine head-to-head drug comparisons and differences between individual drugs in those at high risk of OH.

## Conclusions

A range of commonly prescribed drugs causing sympathetic inhibition are associated with significantly increased odds of OH compared to placebo and should be administered with caution in adults at risk of OH. Most vasodilators were not significantly associated with OH, but this may differ according to dose, age, and high-risk groups. Our study suggests that drugs with combined mechanisms targeting different parts of the orthostatic BP reflex pathway may carry a harmful cumulative risk of OH. Therefore, patients with polypharmacy may be at greatest risk of drug-induced OH and could benefit from routine postural BP monitoring. These results are intended to facilitate safer prescribing choices for an ageing population susceptible to OH and polypharmacy.

## Supporting information

S1 Text Search strategy. (DOCX)

S1 Table Characteristics of studies. (DOCX)

S2 Table Table A. GRADE assessments. Table B. GRADE assessment criteria.
(DOCX)

S1 Fig Risk of bias results. (DOCX)

S2 Fig Subgroup analyses and funnel plots. (DOCX)

S1 Checklist PRISMA guideline. (DOCX)

## Abbreviations

5-ARI: 5-alpha reductase inhibitor
ACE: angiotensin-converting enzyme
ARB: angiotensin receptor blocker
BNF: British National Formulary
BP: blood pressure
BPH: benign prostatic hypertrophy
CCB: calcium channel blocker
GLP-1: glucagon-like peptide-1
GRADE: Grading of Recommendations Assessment, Development, and Evaluation
INN: international nonproprietary name
LUTS: lower urinary tract symptoms
OH: orthostatic hypotension
OR: odds ratio
PD: Parkinson disease
RCT: randomised controlled trial
SGLT-2: sodium–glucose cotransporter-2
SSRI: selective serotonin reuptake inhibitor
T2DM: type 2 diabetes mellitus
