# Supplementary material for: Drug-induced orthostatic hypotension: A systematic review and meta-analysis of randomised controlled trials
Source: PLoS Med. 2021 Nov 9;18(11):e1003821. doi: 10.1371/journal.pmed.1003821 (PMC8577726; doi:10.1371/journal.pmed.1003821)
Supplement: S1 Text — (DOCX) [file pmed.1003821.s001.docx]

**Supplementary Data**

**S1 Text: Search strategy**

| **Database** | **Search strategy** |
| --- | --- |
| MEDLINE | *General medication terms*   1. Hypotension, Orthostatic/ 2. orthostatic hypotension.tw. 3. Orthostatic Intolerance/ 4. orthostatic intolerance.tw. 5. postural hypotension.tw. 6. orthostatic stress.tw. 7. 1 OR 2 OR 3 OR 4 OR 5 OR 6 8. pharmaceutical preparations/ or dosage forms/ or drug combinations/ or drugs, essential/ or drugs, generic/ or drugs, investigational/ or prescription drugs/ 9. pharmaceutical formulation*.tw 10. Drug Delivery Systems/ 11. Fixed dose combination*.tw 12. Drug Therapy/ 13. "Drug-Related Side Effects and Adverse Reactions"/ 14. OR/8-13 15. 7 AND 14 16. randomized controlled trial.pt. 17. controlled clinical trial.pt. 18. randomized.ab. 19. placebo.ab. 20. drug therapy.fs. 21. randomly.ab. 22. trial.ab. 23. groups.ab. 24. OR/15-22 25. exp animals/ not humans.sh. 26. 24 not 25 27. 15 AND 26 |
| MEDLINE | *Individual drug names (automated macro)*   1. orthostatic hypotension.tw. 2. Hypotension, Orthostatic/ 3. orthostatic intolerance.tw. 4. Orthostatic Intolerance/ 5. postural hypotension.tw. 6. orthostatic stress.tw. 7. OR/1-7 8. [BNF DRUG NAME*.mp or MeSH term] AND 7 9. randomized controlled trial.pt. 10. controlled clinical trial.pt. 11. randomized.ab. 12. placebo.ab. 13. drug therapy.fs. 14. randomly.ab. 15. trial.ab. 16. groups.ab. 17. OR/9-16 18. exp animals/ not humans.sh. 19. 17 not 18 20. 8 AND 19 |
| EMBASE | *General medication terms*   1. orthostatic hypotension.tw. 2. orthostatic hypotension/ 3. orthostatic intolerance.tw. 4. orthostatic intolerance/ 5. postural hypotension.tw. 6. orthostatic stress.tw. 7. orthostatic stress/ 8. 1 OR 2 OR 3 OR 4 OR 5 OR 6 OR 7 9. drug/ or essential drug/ or generic drug/ or long acting drug/ or new drug/ or prescription drug/ or short acting drug/ 10. drug therapy/ae [Adverse Drug Reaction] 11. drug combination/ 12. drug effect/ 13. "Drug-Related Side Effects and Adverse Reactions"/ 14. adverse drug reaction/ 15. tablet formulation/ or drug formulation/ 16. drug delivery system/ 17. fixed dose combination*.tw. 18. OR/9-17 19. 8 AND 18 20. random:.tw. 21. clinical trial:.mp. 22. exp health care quality/ 23. 20 OR 21 OR 22 24. 19 AND 23 25. 24 not ((exp animal/ or nonhuman/) not exp human/) |
| EMBASE | *Individual drug names (automated macro program)*   1. orthostatic hypotension.tw. 2. orthostatic hypotension/ 3. orthostatic intolerance.tw. 4. orthostatic intolerance/ 5. postural hypotension.tw. 6. orthostatic stress.tw. 7. orthostatic stress/ 8. 1 OR 2 OR 3 OR 4 OR 5 OR 6 OR 7 9. [DRUG NAME.mp OR MeSH term] AND 8 10. random:.tw. 11. clinical trial:.mp. 12. exp health care quality/ 13. 10 or 11 or 12 14. 9 AND 13 15. 14 not ((exp animal/ or nonhuman/) not exp human/) |
| Web of Science | 1. “orthostatic hypotension” 2. “postural hypotension” 3. “orthostatic intolerance” 4. “orthostatic stress” 5. 1 OR 2 OR 3 OR 4 6. “pharmaceutical preparation*” 7. “dosage form*” 8. “drug combinations*” 9. “pharmaceutical formulation*” 10. “drug delivery system*” 11. “fixed dose combination*” 12. “drug therapy*” 13. "drug side effect*” 14. “drug adverse reaction*” 15. drug* 16. medication* 17. OR/6-16 18. 5 AND 17 |
