# Supplementary material for: Drug-induced orthostatic hypotension: A systematic review and meta-analysis of randomised controlled trials
Source: PLoS Med. 2021 Nov 9;18(11):e1003821. doi: 10.1371/journal.pmed.1003821 (PMC8577726; doi:10.1371/journal.pmed.1003821)
Supplement: S1 Table — (DOCX) [file pmed.1003821.s002.docx]

**S2 Table: Characteristics of Studies**

| **Study name** | **OH*** | **Risk of Bias (RoB 2 score)** | **Study design** | **Drug** | **No. randomised** | **Mean age (SD)** | **% female** | **dose (min-max, mg, frequency)** | **mean dose** | **dose** | **mode** | **duration of admin** | **trial length** | **Recruitment country** | **single/double blinded** | **single/multi centre** | **setting** | **Specific patient group?** | **Placebo run in/wash out** | **sponsored** |
| --- | --- | --- | --- | --- | --- | --- | --- | --- | --- | --- | --- | --- | --- | --- | --- | --- | --- | --- | --- | --- |
| Kennelly 2011[1] | measure + validated | high | parallel | nilvadipine | 86 | 69.3 (8.4) | 48.2 | 8mg | 8mg OD | low | PO | 8 wks | 8 weeks | Ireland | single | single | community | Alzheimer’s disease | n | n |
| de Heus 2019[2] | measured + validated | low | parallel | nilvadipine | 511 | 72.4 (8.6) | 65 | 8mg | 8mg OD | low | PO | 78 wks | 78 weeks | Europe | double | multi | community | mild to moderate AD | n | n |
| Deedwania 1993[3] | measured | high | crossover | amlodipine | 43 | 59 | 14 | 2.5-10mg | 5mg OD | low | PO | 3 wks | 4 weeks | USA | double | single | community | angina | y | y |
| Sramek 1994[4] | bp examined | low | parallel | nimodipine | 61 | 26.5 | 0 | 50mg | 50mg | low | IV | 48 hours | 3 weeks | USA | double | single | community | healthy | y | n |
| Forette 1985[5] | measured + validated | low | parallel | nicardipine | 31 | 86 (2) | 93 | 30-90mg | 69.4mg | low | PO | 4 wks | 5 weeks | France | double | single | community | severe hypertension (>160) | Y | n |
| Sassano 1984[6] | measured + validated | low | parallel | enalapril | 100 | 47.4 | 25 | 20mg | OD | low | PO | 4 wks | 5 weeks | France | double | single | clinic | hypertension | y | n |
| Sumukadas 2018[7] | measured + validated | low | parallel | perindopril | 80 | 78.1 (7.3) | 75 | 2-4mg | 4mg | low | PO | 15 wks | 15 weeks | UK | double | multi | community/GP/clinic | 1x fall; over 65 | n | n |
| Patat 1989[8] | measured + validated | some concerns | sequential | trandolapril | 90 | no info | 0 | 0.125-32mg | (2mg group) | low | PO | 8 days | 5 weeks | France | double | single | community | healthy | y | n |
| Maclean 1988 [9] | measured + validated | some concerns | parallel | quinapril | 270 | 51 | 32 | 20-80mg | 80mg (OD/BD) | high | PO | 12 weeks | 14 weeks | Scotland | double | multi | community | WHO stage I/II htn; non-childbearing | y | n |
| Pool 1999[10] | measured + validated | low | parallel | valsartan | 122 | 54.3 (+10) | 24 | 10-160mg | 10-160mg | low | PO | 4 weeks | 5 weeks | USA | double | multi | community | mild-moderate hypertension | y | n |
| Chrysant 2010[11] | bp examined | some concerns | parallel | olmesartan | 130 | 56.1 (10.1) | 67.9 | 20-40mg | 40mg | high | PO | 12 weeks | 16 weeks | USA | double | multi | community | stage 1 hypertension | y | y |
| Philipp 2011[12] | measured | low | parallel | valsartan | 834 | 57 | 49.7 | 320mg | 320mg | high | PO | 14 weeks | 8 weeks | Europe | double | multi | community | stage 1-2 hypertension | y | y |
| Morrell 2005[13] | measured | some concerns | parallel | losartan | 40 | 67 | 50 | 25mg | 25mg | low | PO | 52 weeks | 48 weeks | UK | double | single | community | COPD | y | y |
| Kasper 2005[14] | measured | low | parallel | escitalopram/fluoxetine | 518 | 75 (7.0) | 75 | 10mg OD/20mg | 10mg OD/20mg | low | PO | 8 weeks | 9 weeks | Europe | double | multi | GP/clinic | depressed, elderly | y | y |
| Kaufman 2008[15] | measured | some concerns | parallel | dapoxetine | 736 | 40.9 (9.71) | 0 | 60mg OD - PRN | 60mg OD | high | PO | 8 weeks | 9 weeks | USA + Canada | double | multi | outpatient | premature ejaculation | y | y |
| Murray 2005[16] | measured | low | parallel | sertraline | 123 | 70.7 (9.7) | 49.4 | 50-100mg | 75mg | low | PO | 26 weeks | 26 weeks | Sweden | double | multi | outpatient | stroke + depression; older | n | n |
| Croft 2014[17] | measured & validated | low | parallel | vilazodone | 518 | 39.3 (12.8) | 51.4 | 10-40mg OD | 30mg | low | PO | 8 weeks | 10 weeks | USA | double | multi | outpatient | major depression | y | y |
| Liebowtiz 2008[18] | measured & validated | some concerns | parallel | desvenlafaxine | 474 | 43 (15) | 62 | 50/100mg OD | 50/100mg | high | PO | 8 weeks | 10 weeks | USA | double | multi | outpatient | major depressive disorder | y | y |
| Raskin 2007[19] | measured & validated | some concerns | parallel | duloxetine | 311 | 72.6 (5.7) | 60.4 | 30-60mg OD | 60mg OD | low | PO | 8 weeks | 10 weeks | USA | double | multi | outpatient | major depressive disorder; older >65 | y | y |
| Lepor 1998[20] | measured | Low | parallel | tamsulosin | 418 |  |  | 0.4mg/0.8mg | 0.4/0.5mg | high | PO | 40 weeks | 40 weeks | USA | double | multi | outpatient | patients with BPH | y | n |
| Giuliano 2006[21] | measured + validated | Low | crossover | Alfuzosin | 18 | 53.1 | 0 | 10 mg OD | 10 mg | high | PO | 2 weeks | 3 weeks | USA | double | single | outpatient | healthy normotensive men | n | y |
| Buzelin 1997[22] | measured + validated | Low | parallel | Alfuzosin | 588 |  |  | 5mg BD (SR) | 10mg | high | PO | 3 months | 3 months | Belgium, France, Germany, Holland & Denmark | double | multi | outpatient | 6 month duration of LUTS related to BPH | y | n |
| Novara 2014 [23] | measured + validated | Low | parallel | Silodosin | 1494 | 64.9 | 0 | 8mg OD | 8mg | high | PO | 12 weeks | 16 weeks | USA, Europe | double | multi | outpatient | ≥50 years, moderate-to-severe LUTS | y | n |
| Morganroth 2010  [24] | BP examined | low | parallel | Silodosin | 186 | 28 | 0 | 8mg & 24mg OD | 8mg/24mg | high | PO | 5 days | 5 days | USA | double | single | outpatient | healthy men aged 18–45 | y | y |
| Marks 2003[25] | measured + validated | Low | crossover | Alfuzosin | 49 | 65.6 | 0 | 10 mg OD | 10mg | high | PO | 21 days | 35 days | USA | single | multi | outpatient | men over 50 with enlarged prostate with BPH (only good complaince accepted) | y | y |
| Lepor 1992[26] | BP examined | low | parallel | terazosin | 285 | 61.8 | 0 | 2, 5 or 10 mg OD | 10 mg | high | PO | 12 weeks | 16 weeks | USA | double | multi | outpatient | men 50-75 with BPH | y | n |
| Lepor 2000[27] | measured + validated | Low | parallel | terazosin | 305 | 65 | 0 | 10 mg OD | 10 mg | high | PO | 2 weeks | 2 weeks | USA | double | multi | outpatient | men with BPH | y | y |
| Yasukawa 2001-1 [28] | BP examined | low | parallel | Tamsulosin | 735 | 57.3 | 0 | 0.4mg/0.8mg OD |  | high | PO | 13 weeks | 17 weeks | USA | double | multi | outpatient | men over 45 yrs with BPH | y | n |
| Yasukawa 2001-2 [28] | BP examined | low | parallel | Tamsulosin | 735 | 58.6 | 0 | 0.4mg/0.8mg OD |  | high | PO | 13 weeks | 17 weeks | USA | double | multi | outpatient | men over 45 yrs with BPH | y | n |
| Roehrborn 2005[29] | measured + validated | Low | parallel | Doxazosin | 213 | 63.5 | 0 | 4mg/day | 4mg | low | PO | 2 weeks | 4 weeks | USA | double | multi | outpatient | 50-80yrs with BPH | y | n |
| Resnick 2007[30] | measured | Low | parallel | Alfuzosin | 372 | 63.5 | 0 | 10mg OD | 10mg | high | PO | 28 days | 28 days | USA | double | multi | outpatient | men with BPH | y | y |
| Djavan 2005[31] | measured | Low | parallel | Tamsulosin | 117 | 66.8 | 0 | 0.4 mg OD | 0.4mg | high | PO | 8 weeks | 10 weeks | Europe | double | multi | outpatient | Men with LUTS suggestive of BPH | y | n |
| Al-Ansari 2009 [32] | BP examined | Low | parallel | Tamsulosin | 100 | 37.18 | 67 | 0.4 mg OD | 0.4mg | high | PO | 4 weeks | 4 weeks | Qatar | double | multi | outpatient | patients with ureteral stones | n | n |
| Andersen 2000[33] | BP examined | Low | parallel | Doxazosin | 795 | 64.9 | 0 | 4mg / 8mg OD |  | high | PO | 13 weeks | 15 weeks | Denmark, Norway, and Sweden | double | multi | outpatient | men between 50 and 80 years with BPH | y | y |
| Singh 2014[34] | measured + validated | Low | parallel | tamsulosin | 60 | 32.7 | 56.7 | 0.4 mg OD | 0.4mg | high | PO | 4 weeks | 4 weeks | USA | single | single | hospital | Patients aged 18–80 years, with urological disorders undergoing ureteral stenting | n | n |
| Chapple 1997[35] | Measured | high | Parallel (pooled 2 RCTs) | Tamsulosin | 627 | 63.6 (4.7) | 0 | 0.4mg OD | 0.4mg | high | PO | 14  weeks | 12 weeks | Europe | double | multi | outpatient | Men with LUTS suggested of BPH | y | y |
| Cohn 2003[36] | BP  examined | low | parallel | Moxonidine | 1934 | 64.1 (11.26) | 23.4 | 0.25-1.5mg | 1.5mg | high | PO | 12 months | 12 months | USA/EU | double | multi | clinic | heart failure; NYHA ii-iv | n | y |
| Evans 1988[37] | measured | some concerns | parallel | methyldopa | 25 | 63.8 (6.4) | 32 | 750-1500mg | 750mg | low | PO | 12 months | 12 months | UK | double | single | clinic | cor pulmonale; copd | n | n |
| Hov 2018[38] | measured + validated | some concerns | parallel | clonidine | 20 | 86 | 65 | 75mcg (plasma) | 75mcg | low | PO | 7 days | 4 months | Norway | double | single | inpatient | delirium, acutely admitted | n | n |
| McIntyre 2004[39] | Measured | low | parallel | Quetiapine/haloperidol | 302 | 42.8 | 64 | 100-800 mg/ 2-8mg | 800 mg/8mg | high | PO | 12 weeks | 15 months | South America / Asia /Europe | double | multi | hospital | Mania associated bipolar I disorder | n | y |
| Kramer 2010[40] | Measured + validated | low | parallel | Paliperidone | 79 | 9.8 | 35 | 150mg TDS | 150mg | low | IM | 64 days | 10 months | USA / Russia / Bulgaria /Poland /Ukraine /India | double | multi | hospital | Schizophrenia patients | y | y |
| Berwaerts, 2012[41] | Measured + Validated | Some concerns | parallel | Paliperidone | 236 | 40 (12.6) | 55 | 3-12 mg | 9mg | low | PO | 12 weeks | 4 years | 17 countries | double | multi |  | Patients currently diagnosed with maniac or mixed episodes | y | y |
| Mallikaarjun , 2004[42] | Measured +validated | Low | parallel | Aripiprazole | 39 |  | 0 | 5-30 mg | 15mg | low | PO | 20 days |  |  | double | single | clinic | Normal Healthy volunteers | n | n |
| Cutler, 2008[43] | Measured + validated | low | parallel | Ziprasidone/iloperidone | 381 | 40 (9.9) | 24.2 | 20-80 mg/1-12mg | 40/12 | low | PO | 28 days | 10 months | USA / India | double | multi | hospital | Patients with acute exacerbations of scizophrenia | n | y |
| Kane, 2007[44] | Measured | low | Parallel | Paliperidone/olanzapine | 413 | 37 (10.2) | 50 | 6-12mg/10mg |  | low | PO | 6 weeks | 10 months | Europe / India | double | multi | hospital | Schizophrenia patients | y | y |
| Miceli, 2000[45] | Measured | Some concerns | Parallel | Ziprasidone | 39 |  | 0 | 40-120mg | 40mg | low | PO | 14 weeks |  |  | double | multi |  | Healthy male volunteers | y | y |
| Tohen, 2003[46] | Measured +Validated | low | Parallel | Olanzapine | 788 | 42.2 (12.5) | 0.63 | 5-20 mg /day | 9.7 mg | low | PO | 8 weeks | 18 months | 13 countries | double | multi | Inpatient/Outpatient | Bipolar I depression | y | n |
| Hough 2010 [47] | Measured | low | Parallel | Palperidone/quetiapine | 94 | 37 (9) | 28 | 12-18 mg/200-800mg |  | high | PO | 10 days | 5 months | USA | double | multi |  | Schizophrenia patients | y | y |
| Carre 1982[48] | measured + validated | low | parallel | labetalol | 25 | 32.45 (12.51) | 10 | 400mg | 400mg | low | PO | 4 weeks | 4 weeks | France | double | single | community | moderate hypertension | n | n |
| Quyyumi 1985[49] | BP  examined | some concerns | crossover | labetalol | 10 | 42-72 years | 20 | 200-600mg | 480mg | low | PO | 2 weeks | 4 weeks | UK | double | single | clinic | stable angina | n | n |
| Cohen-Solal 2005[50] | BP  examined | some concerns | parallel | carvedilol | 54 | 59 (12) | 14 | 3.25-25mg | 25mg | low | PO | 6 months | 6 months | France | double | multi | clinic | chronic heart failure* | n | y |
| Zacharias 1970[51] | measured | low | crossover | propranolol | 28 | 51.5 | 39 | 120-320mg | 250mg | low | PO | 16 weeks | 32 weeks | UK | double | single | GP | hypertension, stable | n | y |
| Greathouse 2010[52] | BP  examined | some concerns | parallel | nebivolol | 811 | 53.9 (11.1) | 46.3 | 5/10/20mg | 10mg | high | PO | 12 weeks | 18 weeks | USA/EU | double | multi | clinic | stage 1-2 hypertension |  | y |
| Louis 1987[53] | measured + validated | low | sequential | carvedilol | 8 | 61 (2) | 0 | 25-50mg/200mg |  | low | PO | 2 days | 14 weeks | Australia | double | single | community | healthy | n | y |
| Krum 1994[54] | measured | some concerns | crossover | carvedilol | 16 | 70 | 0 | 12.5-25mg | 25mg | low | PO | 1 day | 4 weeks | Australia | double | single | community | older; mild/mod hypertension | y | y |
| Davidov 1983[55] | measured + validated | some concerns | parallel | labetalol | 74 | 55 | 50 | 200-1200mg | 600mg | low | PO | 4 weeks | 17 weeks | USA | double | single | community | mild hypertension | y | n |
| Georgotas 1987[56] | measured & validated | low | parallel | nortriptyline | 75 | >55 (no info) | no info | 50-170ng/ml | 79mg OD | low | PO | 7 weeks | 9 weeks | USA | double | single | outpatient | major depressive disorder | y | n |
| Neshkes 1985[57] | measured & validated | high | parallel | imipramine/doxepin | 39 | 66.9 (7.0) | 86 | 25-250mg/25-225mg | 83.3mg/76.1 OD | low | PO | 4-36 weeks | 36 weeks | USA | double | single | outpatient | major depressive episode >2 months; >55 | y | n |
| Claghorn 1983[58] | measured | low | parallel | amitriptyline | 172 | 39.2 | 57 | 25-180mg OD | 180mg | high | PO | 4 weeks | 5 weeks | US | double | multi | outpatient | major depressive disorder | y | n |
| Sjostrom 2015 (1) [59] | measured + validated | Some concerns | 13 parallel (pooled) | dapagliflozin | 3223 | 58 (20-84) | 44.2 | 10mg OD | 10mg OD | high | PO | 24 weeks | 24 weeks | Europe | double | multi | outpatient | T2DM (poor glycaemic control) + HTN | n | y |
| Sjostrom 2015 (2) [59] | measured + validated | Some concerns | parallel | dapagliflozin | 1293 |  |  | 10mg OD | 10mg OD | high | PO |  |  |  | double | multi |  | T2DM only | n |  |
| Leiter 2016 (LT1)[60] | measured | some concerns | 2 parallel RCTs pooled (extension) | dapagliflozin | 1649 | 63.4 (7.4) | 32.6 | 10mg OD | 10mg OD | high | PO | 2yrs +24 wks | 24 weeks | Can/USA/Europe | double | multi | outpatient | T2DM; high CVD risk | n | y |
| Cefalu 2015[61] | measured | high | parallel | dapagliflozin | 922 | 62.8 (7) | 32.1 | 10mg OD | 10mg OD | high | PO | 52 weeks | 52 weeks | Can/USA/Europe | double | multi | outpatient | T2DM; high CVD risk | n | y |
| Sha 2011[62] | measured | high | parallel | canagliflozin | 63 | 37.1 (19-55) | 0 | 100-800mg | 400mg OD | high | PO | 1 week | 8 days | USA | double | single | community | healthy men | n | y |
| Stenlof 2013[63] | measured | some concerns | parallel | canagliflozin | 584 | 55.3 (10.2) | 54.8 | 300mg | 100mg/300mg OD | high | PO | 26 weeks | 34 weeks | 17 countries | double | multi | outpatient | T2DM hba1c 7-10% | y | y |
| Tikkanen 2015[64] | measured + validated | low | parallel | empagliflozin | 825 | 59.9 (9.7) | 43.5 | 10/25mg | 10/25mg OD | high | PO | 12 weeks | 12 weeks | USA/Eu/Middle East | double | multi | outpatient | T2DM + HTN | n | y |
| Yale 2013[65] | BP  examined | Some concerns | parallel | canagliflozin | 272 | 69.5 (8.2) | 32 | 100/300mg | 100/300mg | High | PO | 52 weeks | 26 weeks | Canada, Aus, US, Europe | double | multi | outpatient | T2DM (hba1c 7-10.5%) | y | y |
| Bode 2013[66] | BP examined | Some concerns | parallel | canagliflozin | 716 | 64.3 (6.5) | 48.5 | 100/300mg | 100/300mg | high | PO | 26 wks | 26 wks | 17 countries | double | multi | outpatient | T2DM (hba1c 7-10.5%) | y | y |
| Bailey 2014[67] | BP examined | high | parallel | dapagliflozin | 485 | 53.0 (11.7) | 44.6 | 2.5/5/10mg | 2.5/5/10mg | high | PO | 102 wks | 102wks | US, Canada, Mexico, Russia | double | multi | Outpatient | T2DM (7-10%) | y | y |
| Other drugs (not eligible for meta-analysis) [68-96] | | | | | | | | | | | | | | | | | | | | |

**OH reporting has been categorised according to: (1) “measured and validated” (a documented postural BP examination performed using a threshold of ≥20 mmHg systolic / ≥10 mm Hg diastolic reduction); (2) “measured” (a documented postural BP examination performed without a specified threshold); (3) “BP examined” (a documented BP examination only – but implied as postural, since the study reports OH)*

1. Kennelly SP, Abdullah L, Paris D, et al. Demonstration of safety in Alzheimer's patients for intervention with an anti-hypertensive drug Nilvadipine: results from a 6-week open label study. Int J Geriatr Psychiatry 2011;**26**(10):1038-45 doi: https://dx.doi.org/10.1002/gps.2638[published Online First: Epub [Accessed 15 September 2021].

2. de Heus RAA, Donders R, Santoso AMM, et al. Blood Pressure Lowering With Nilvadipine in Patients With Mild-to-Moderate Alzheimer Disease Does Not Increase the Prevalence of Orthostatic Hypotension. J Am Heart Assoc 2019;**8**(10) doi: 10.1161/jaha.119.011938[published Online First: Epub Date] [Accessed 15 September 2021]|.

3. Deedwania PC, Cheitlin MD, Das SK, Pool PE, Singh JB, Pasternak RC. Amlodipine once a day in stable angina: double-blind crossover comparison with placebo. Clin Cardiol 1993;**16**(8):599-602

4. Sramek JJ, Heller AH, Sundaresan PR, Lettieri J, Sawin S, Cutler NR. Safety and tolerance of intravenous nimodipine. Ann Pharmacother 1994;**28**(10):1143-8

5. Forette F, Bellet M, Henry JF, Hervy MP, Poyard-Salmeron C, Bouchacourt P. [Treatment of arterial hypertension in the aged with a calcium antagonist: nicardipine]. Arch Mal Coeur Vaiss 1984;**77**(11):1242-6

6. Sassano P, Chatellier G, Alhenc-Gelas F. Antihypertensive effect of enalapril as first-step treatment of mild and moderate uncomplicated essential hypertension. Evaluation by two methods of blood pressure measurement. Am J Med 1984;**77**(2 A):18-22

7. Sumukadas D, Price R, McMurdo MET, et al. The effect of perindopril on postural instability in older people with a history of falls-a randomised controlled trial. Age Ageing 2018;**47**(1):75-81 doi: https://dx.doi.org/10.1093/ageing/afx127[published Online First: Epub Date]| [Accessed 15 September 2021].

8. Patat A, Surjus A, Le Go A, Granier J. Safety and tolerance of single oral doses of trandolapril (RU 44.570), a new angiotensin converting enzyme inhibitor. Eur J Clin Pharmacol 1989;**36**(1):17-23

9. Maclean D. Quinapril: a double-blind, placebo-controlled trial in essential hypertension. Angiology 1989;**40**(4 Pt 2):370-81

10. Pool JL, Glazer R, Chiang YT, Gatlin M. Dose-response efficacy of valsartan, a new angiotensin II receptor blocker. J Hum Hypertens 1999;**13**(4):275-81

11. Chrysant SG. Safety and tolerability of an olmesartan medoxomil-based regimen in patients with stage 1 hypertension: a randomized, double-blind, placebo-controlled study. Clin Drug Invest 2010;**30**(7):473-82 doi: https://dx.doi.org/10.2165/11536560-000000000-00000[published Online First: Epub Date]|[Accessed 15 September 2021].

12. Philipp T, Glazer RD, Wernsing M, Yen J. Initial combination therapy with amlodipine/valsartan compared with monotherapy in the treatment of hypertension. J Am Soc Hypertens 2011;**5**(5):417-24 doi: 10.1016/j.jash.2011.02.008[published Online First: Epub Date] [Accessed 15 September 2021]|.

13. Morrell NW, Higham MA, Phillips PG, Shakur BH, Robinson PJ, Beddoes RJ. Pilot study of losartan for pulmonary hypertension in chronic obstructive pulmonary disease. Respiratory Research 2005;**6 (no pagination)**

14. Kasper S, De Swart H, Andersen HF. Escitalopram in the treatment of depressed elderly patients. Am J Geriatr Psychiatry 2005;**13**(10):884-91

15. Kaufman JM, Rosen RC, Mudumbi RV, Tesfaye F, Hashmonay R, Rivas D. Treatment benefit of dapoxetine for premature ejaculation: Results from a placebo-controlled phase III trial. BJU Int 2009;**103**(5):651-58

16. Murray V, Von Arbin M, Bartfai A, et al. Double-blind comparison of sertraline and placebo in stroke patients with minor depression and less severe major depression. J Clin Psychiatry 2005;**66**(6):708-16

17. Croft HA, Pomara N, Gommoll C, Chen D, Nunez R, Mathews M. Efficacy and safety of vilazodone in major depressive disorder: A randomized, double-blind, placebo-controlled trial. J Clin Psychiatry 2014;**75**(11):e1291-e98

18. Liebowitz MR, Manley AL, Padmanabhan SK, Ganguly R, Tummala R, Tourian KA. Efficacy, safety, and tolerability of desvenlafaxine 50 mg/day and 100 mg/day in outpatients with major depressive disorder. Current Medical Research and Opinion 2008;**24**(7):1877-90

19. Raskin J, Wiltse CG, Siegal A, et al. Efficacy of duloxetine on cognition, depression, and pain in elderly patients with major depressive disorder: An 8-week, double-blind, placebo-controlled trial. Am J Psychiatry 2007;**164**(6):900-09

20. Lepor H, Tamsulosin Investigator G. Long-term evaluation of tamsulosin in benign prostatic hyperplasia: Placebo-controlled, double-blind extension of Phase III trial. Urology 1998;**51**(6):901-06 doi: 10.1016/s0090-4295(98)00127-7[published Online First: Epub Date]| [Accessed 15 September 2021].

21. Giuliano F, Kaplan SA, Cabanis MJ, Astruc B. Hemodynamic interaction study between the alpha1-blocker alfuzosin and the phosphodiesterase-5 inhibitor tadalafil in middle-aged healthy male subjects. Urology 2006;**67**(6):1199-204

22. Buzelin JM, Fonteyne E, Kontturi M, Witjes WPJ, Khan A. Comparison of tamsulosin with alfuzosin in the treatment of patients with lower urinary tract symptoms suggestive of bladder outlet obstruction (symptomatic benign prostatic hyperplasia). British Journal of Urology 1997;**80**(4):597-605

23. Novara G, Chapple CR, Montorsi F. A pooled analysis of individual patient data from registrational trials of silodosin in the treatment of non-neurogenic male lower urinary tract symptoms (LUTS) suggestive of benign prostatic hyperplasia (BPH). BJU Int 2014;**114**(3):427-33 doi: 10.1111/bju.12712[published Online First: Epub Date]|.

24. Morganroth J, Lepor H, Hill LA, Volinn W, Hoel G. Effects of the selective alpha 1a-adrenoceptor antagonist silodosin on ECGs of healthy men in a randomized, double-blind, placebo-and moxifloxacin-controlled study. Clinical Pharmacology and Therapeutics 2010;**87**(5):609-13

25. Marks LS, Roehrborn CG, Gittelman M, Kim D, Forrest J, Jacobs S. First dose efficacy of alfuzosin once daily in men with symptomatic benign prostatic hyperplasia. Urology 2003;**62**(5):888-93

26. Lepor H, Henry D, Laddu AR. The efficacy and safety of terazosin for the treatment of symptomatic BPH. Prostate 1991;**18**(4):345-55

27. Lepor H, Jones K, Williford W. The mechanism of adverse events associated with terazosin: an analysis of the Veterans Affairs cooperative study. J Urol 2000;**163**(4):1134-7

28. Yasukawa K, Swarz H, Ito Y. Review of orthostatic tests on the safety of tamsulosin, a selective alpha1A-adrenergic receptor antagonist, shows lack of orthostatic hypotensive effects. J Int Med Res 2001;**29**(3):236-51

29. Roehrborn CG. Alfuzosin 10 mg once daily prevents overall clinical progression of benign prostatic hyperplasia but not acute urinary retention: Results of a 2-year placebo-controlled study. BJU Int 2006;**97**(4):734-41

30. Resnick MI, Roehrborn CG. Rapid onset of action with alfuzosin 10 mg once daily in men with benign prostatic hyperplasia: A randomized, placebo-controlled trial. Prostate Cancer and Prostatic Diseases 2007;**10**(2):155-59

31. Djavan B, Chapple C, Milani S, Marberger M. State of the art on the efficacy and tolerability of alpha <inf>1</inf>-adrenoceptor antagonists in patients with lower urinary tract symptoms suggestive of benign prostatic hyperplasia. Urology 2004;**64**(6):1081-88

32. Al-Ansari A, Al-Naimi A, Alobaidy A, Assadiq K, Azmi MD, Shokeir AA. Efficacy of Tamsulosin in the Management of Lower Ureteral Stones: A Randomized Double-blind Placebo-controlled Study of 100 Patients. Urology 2010;**75**(1):4-7

33. Andersen M, Dahlstrand C, Hoye K. Double-blind trial of the efficacy and tolerability of doxazosin in the gastrointestinal therapeutic system, doxazosin standard, and placebo in patients with benign prostatic hyperplasia. Eur Urol 2000;**38**(4):400-09

34. Singh I, Tripathy S, Agrawal V. Efficacy of tamsulosin hydrochloride in relieving "double-J ureteral stent-related morbidity": a randomized placebo controlled clinical study. International Urology and Nephrology 2014;**46**(12):2279-83

35. Chapple CR, Baert L, Thind P, Hofner K, Khoe GSS, Spangberg A. Tamsulosin 0.4 mg once daily: Tolerability in older and younger patients with lower urinary tract symptoms suggestive of benign prostatic obstruction (symptomatic BPH). Eur Urol 1997;**32**(4):462-70

36. Cohn JN, Pfeffer MA, Rouleau J, et al. Adverse mortality effect of central sympathetic inhibition with sustained-release moxonidine in patients with heart failure (MOXCON). European Journal of Heart Failure 2003;**5**(5):659-67

37. Evans TW, Waterhouse J, Finlay M, Suggett AJ, Howard P. The effects of long term methyldopa in patients with hypoxic cor pulmonale. Br J Dis Chest 1988;**82**(4):405-13

38. Hov KR, Neerland BE, Andersen AM, et al. The use of clonidine in elderly patients with delirium; pharmacokinetics and hemodynamic responses. BMC Pharmacology and Toxicology 2018;**19**(1)

39. McIntyre RS, Brecher M, Paulsson B, Huizar K, Mullen J. Quetiapine or haloperidol as monotherapy for bipolar mania - A 12-week, double-blind, randomised, parallel-group, placebo-controlled trial. Eur Neuropsychopharmacol 2005;**15**(5):573-85

40. Kramer M, Litman R, Hough D, et al. Paliperidone palmitate, a potential long-acting treatment for patients with schizophrenia. Results of a randomized, double-blind, placebo-controlled efficacy and safety study. International Journal of Neuropsychopharmacology 2010;**13**(5):635-47

41. Berwaerts J, Cleton A, Rossenu S, et al. A comparison of serum prolactin concentrations after administration of paliperidone extended-release and risperidone tablets in patients with schizophrenia. J Psychopharmacol 2010;**24**(7):1011-8 doi: https://dx.doi.org/10.1177/0269881109106914[published Online First: Epub Date]|[Accessed 15 September 2021].

42. Mallikaarjun S, Salazar DE, Bramer SL. Pharmacokinetics, Tolerability, and Safety of Aripiprazole following Multiple Oral Dosing in Normal Healthy Volunteers. J Clin Pharmacol 2004;**44**(2):179-87

43. Cutler AJ, Kalali AH, Weiden PJ, Hamilton J, Wolfgang CD. Four-week, double-blind, placebo- and ziprasidone-controlled trial of lloperidone in patients with acute exacerbations of schizophrenia. J Clin Psychopharmacol 2008;**28**(2):S20-S28 doi: 10.1097/JCP.0b013e318169d4ce[published Online First: Epub Date]| [Accessed 15 September 2021].

44. Kane J, Canas F, Kramer M, et al. Treatment of schizophrenia with paliperidone extended-release tablets: A 6-week placebo-controlled trial. Schizophr Res 2007;**90**(1-3):147-61

45. Miceli JJ, Wilner KD, Hansen RA, Johnson AC, Apseloff G, Gerber N. Single- and multiple-dose pharmacokinetics of ziprasidone under non-fasting conditions in healthy male volunteers. Br J Clin Pharmacol 2000;**49**:5S-13S

46. Tohen M, Vieta E, Calabrese J, et al. Efficacy of Olanzapine and Olanzapine-Fluoxetine Combination in the Treatment of Bipolar I Depression. Arch Gen Psychiatry 2003;**60**(11):1079-88

47. Hough DW, Natarajan J, Vandebosch A, Rossenu S, Kramer M, Eerdekens M. Evaluation of the effect of paliperidone extended release and quetiapine on corrected QT intervals: A randomized, double-blind, placebo-controlled study. Int Clin Psychopharmacol 2011;**26**(1):25-34

48. Carre AG, Bertrand ME, Libersa CC, Lekieffre JP. Efficacy and tolerance of labetalol 400 mg/day in moderate essential hypertension in adults: A double blind trial against placebo. [French]. Rev Med Interne 1982;**3**(4):373-77

49. Quyyumi AA, Wright C, Mockus L. Effects of combined alpha and beta adrenoceptor blockade in patients with angina pectoris. A double blind study comparing labetalol with placebo. Br Heart J 1985;**53**(1):47-52

50. Cohen-Solal A, Rouzet F, Berdeaux A, et al. Effects of carvedilol on myocardial sympathetic innervation in patients with chronic heart failure. J Nucl Med 2005;**46**(11):1796-803

51. Zacharias FJ, Cowen KJ. Controlled trial of propranolol in hypertension. Br Med J 1970;**1**(5694):471-4

52. Greathouse M. Nebivolol efficacy and safety in patients with stage I-II hypertension. Clin Cardiol 2010;**33**(4):E20-E27

53. Louis WJ, McNeil JJ, Workman BS, Drummer OH, Conway EL. A pharmacokinetic study of carvedilol (BM 14.190) in elderly subjects: preliminary report. J Cardiovasc Pharmacol 1987;**10 Suppl 11**:S89-93

54. Krum H, Conway EL, Broadbear JH, Howes LG, Louis WJ. Postural hypotension in elderly patients given carvedilol. Bmj 1994;**309**(6957):775-6

55. Davidov ME, Moir GD, Poland MP, Maloy J, Medakovic M. Monotherapy with labetalol in the treatment of mild hypertension: A double-blind study. Am J Med 1983;**75**(4 A):47-53

56. Georgotas A, McCue RE, Friedman E, Cooper TB. A placebo-controlled comparison of the effect of nortriptyline and phenelzine on orthostatic hypotension in elderly depressed patients. J Clin Psychopharmacol 1987;**7**(6):413-6

57. Neshkes RE, Gerner R, Jarvik LF, et al. Orthostatic effect of imipramine and doxepin in depressed geriatric outpatients. J Clin Psychopharmacol 1985;**5**(2):102-6

58. Claghorn J, Gershon S, Goldstein BJ. Zimelidine tolerability in comparison to amitriptyline and placebo: Findings from a multicentre trial. Acta Psychiatr Scand 1983;**68**(SUPPL. 308):104-14

59. Sjostrom CD, Johansson P, Ptaszynska A, List J, Johnsson E. Dapagliflozin lowers blood pressure in hypertensive and non-hypertensive patients with type 2 diabetes. Diabetes and Vascular Disease Research 2015;**12**(5):352-58

60. Leiter LA, Cefalu WT, de Bruin TWA, et al. Long-term maintenance of efficacy of dapagliflozin in patients with type 2 diabetes mellitus and cardiovascular disease. Diabetes, Obesity and Metabolism 2016;**18**(8):766-74

61. Cefalu WT, Leiter LA, de Bruin TW, Gause-Nilsson I, Sugg J, Parikh SJ. Dapagliflozin's Effects on Glycemia and Cardiovascular Risk Factors in High-Risk Patients With Type 2 Diabetes: A 24-Week, Multicenter, Randomized, Double-Blind, Placebo-Controlled Study With a 28-Week Extension. Diabetes Care 2015;**38**(7):1218-27 doi: 10.2337/dc14-0315[published Online First: Epub Date]| [Accessed 15 September 2021].

62. Sha S, Devineni D, Ghosh A, et al. Canagliflozin, a novel inhibitor of sodium glucose co-transporter 2, dose dependently reduces calculated renal threshold for glucose excretion and increases urinary glucose excretion in healthy subjects. Diabetes, Obesity and Metabolism 2011;**13**(7):669-72

63. Stenlof K, Cefalu WT, Kim KA, et al. Efficacy and safety of canagliflozin monotherapy in subjects with type 2 diabetes mellitus inadequately controlled with diet and exercise. Diabetes, Obesity and Metabolism 2013;**15**(4):372-82

64. Tikkanen I, Narko K, Zeller C, et al. Empagliflozin reduces blood pressure in patients with type 2 diabetes and hypertension. Diabetes Care 2015;**38**(3):420-8 doi: 10.2337/dc14-1096[published Online First: Epub Date]| [Accessed 15 September 2021].

65. Yale JF, Bakris G, Cariou B, et al. Efficacy and safety of canagliflozin over 52 weeks in patients with type 2 diabetes mellitus and chronic kidney disease. Diabetes Obes Metab 2014;**16**(10):1016-27 doi: 10.1111/dom.12348[published Online First: Epub Date]|.

66. Bode B, Stenlof K, Harris S, et al. Long-term efficacy and safety of canagliflozin over 104 weeks in patients aged 55-80 years with type 2 diabetes. Diabetes Obes Metab 2015;**17**(3):294-303 doi: 10.1111/dom.12428[published Online First: Epub Date]|.

67. Bailey CJ, Morales Villegas EC, Woo V, Tang W, Ptaszynska A, List JF. Efficacy and safety of dapagliflozin monotherapy in people with Type 2 diabetes: a randomized double-blind placebo-controlled 102-week trial. Diabet Med 2015;**32**(4):531-41 doi: 10.1111/dme.12624[published Online First: Epub Date]|.

68. Bohm M, Burkart M, Baumann G. Sildenafil is well tolerated by erectile dysfunction patients taking antihypertensive medications, including those on multidrug regimens. Curr Drug Saf;**2**(1):5-8

69. Packer M, McMurray J, Massie BM, et al. Clinical effects of endothelin receptor antagonism with bosentan in patients with severe chronic heart failure: Results of a pilot study. Journal of Cardiac Failure;**11**(1):12-20

70. Jorsal A, Kistorp C, Holmager P, et al. Effect of liraglutide, a glucagon-like peptide-1 analogue, on left ventricular function in stable chronic heart failure patients with and without diabetes (LIVE)-a multicentre, double-blind, randomised, placebo-controlled trial. European Journal of Heart Failure;**19**(1):69-77

71. Wallace MS, Charapata SG, Fisher R, et al. Intrathecal ziconotide in the treatment of chronic nonmalignant pain: A randomized, double-blind, placebo-controlled clinical trial. Neuromodulation;**9**(2):75-86

72. Pahwa R, Koller WC, Trosch RM, Sherry JH, Investigators APOS. Subcutaneous apomorphine in patients with advanced Parkinson's disease: A dose-escalation study with randomized, double-blind, placebo-controlled crossover evaluation of a single dose. J Neurol Sci;**258**(1):137-43

73. Swift CG, Lee DR, Maskrey VL, Yisak W, Jackson SHD, Tiplady B. Single dose pharmacodynamics of thioridazine and remoxipride in healthy younger and older volunteers. J Psychopharmacol 1999;**13**(2):159-65

74. Fishman M, Tirado C, Alam D, Gullo K, Clinch T, Gorodetzky CW. Safety and Efficacy of Lofexidine for Medically Managed Opioid Withdrawal: A Randomized Controlled Clinical Trial. Journal of Addiction Medicine;**13**(3):169-76

75. Hauser RA, Isaacson SH, Ellenbogen A, et al. Orally inhaled levodopa (CVT-301) for early morning OFF periods in Parkinson's disease. Parkinsonism and Related Disorders;**64**:175-80

76. Limousin P, Pollak P, Pfefen JP, Tournier-Gervason CL, Dubuis R, Perret JE. Acute administration of levodopa-benserazide and tolcapone, a COMT inhibitor, in Parkinson's disease. Clin Neuropharmacol 1995;**18**(3):258-65

77. Tran JQ, Hartung JP, Olson AD, et al. Cardiac Safety of Ozanimod, a Novel Sphingosine-1-Phosphate Receptor Modulator: Results of a Thorough QT/QTc Study. Clinical Pharmacology in Drug Development;**7**(3):263-76

78. Jansen RW, Van Lier HJ, Hoefnagels WH. Nitrendipine versus hydrochlorothiazide in hypertensive patients over 70 years of age. Clin Pharmacol Ther;**45**(3):291-8

79. Hauser RA, Hubble JP, Truong DD. Randomized trial of the adenosine A<inf>2A</inf> receptor antagonist istradefylline in advanced PD. Neurology;**61**(3):297-303

80. Bleiberg H, Gerard B, Dalesio O, Crespeigne N, Rozencweig M. Activity of a new antiemetic agent: alizapride. A randomized double-blind crossover controlled trial. Cancer Chemother Pharmacol 1988;**22**(4):316-20

81. Chalmers J, Castaigne A, Morgan T, Chastang C. Long-term efficacy of a new, fixed, very-low-dose angiotensin-converting enzyme-inhibitor/diuretic combination as first-line therapy in elderly hypertensive patients. J Hypertens 2000;**18**(3):327-37

82. Ma A, Garl, WT, et al. A pilot study of ranolazine in patients with intermittent claudication. Int Angiol;**25**(4):361-69

83. Hussain IF, Brady CM, Swinn MJ, Mathias CJ, Fowler CJ. Treatment of erectile dysfunction with sildenafil citrate (Viagra) in parkinsonism due to Parkinson's disease or multiple system atrophy with observations on orthostatic hypotension. J Neurol Neurosurg Psychiatry;**71**(3):371-4

84. Zeltser D, Rosansky S, Van Rensburg H, Verbalis JG, Smith N. Assessment of the efficacy and safety of intravenous conivaptan in euvolemic and hypervolemic hyponatremia. Am J Nephrol;**27**(5):447-57

85. LaCroix AZ, Ott SM, Ichikawa L, Scholes D, Barlow WE. Low-dose hydrochlorothiazide and preservation of bone mineral density in older adults: A randomized, double-blind, placebo-controlled trial. Ann Intern Med;**133**(7):516-26

86. Takahashi M, Yuasa R, Imai T, et al. Selegiline (L-deprenyl) and L-dopa treatment of Parkinson's disease: A double-blind trial. Intern Med 1994;**33**(9):517-24

87. Amsterdam JD, Bodkin JA. Selegiline transdermal system in the prevention of relapse of major depressive disorder: A 52-week, double-blind, placebo-substitution, parallel-group clinical trial. J Clin Psychopharmacol;**26**(6):579-86

88. Moller JC, Oertel WH, Koster J, Pezzoli G, Provinciali L. Long-term efficacy and safety of pramipexole in advanced Parkinson's disease: Results from a European multicenter trial. Mov Disord;**20**(5):602-10

89. Larsen JR, Vedtofte L, Jakobsen MSL, et al. Effect of liraglutide treatment on prediabetes and overweight or obesity in clozapine- or olanzapine-treated patients with schizophrenia spectrum disorder: A randomized clinical trial. JAMA Psychiatry;**74**(7):719-28

90. Miller PD, Hattersley G, Riis BJ, et al. Effect of abaloparatide vs placebo on newvertebral fractures in postmenopausalwomen with osteoporosis a randomized clinical trial. JAMA - Journal of the American Medical Association;**316**(7):722-33

91. Bruce RA, Hossack KF, Kusumi F, Day B, Kannagi T. Excessive reduction in peripheral resistance during exercise and risk of orthostatic symptoms with sustained-release nitroglycerin and diltiazem treatment of angina. Am Heart J;**109**(5):1020-6

92. Pahwa R, Stacy MA, Factor SA, et al. Ropinirole 24-hour prolonged release: randomized, controlled study in advanced Parkinson disease. Neurology;**68**(14):1108-15

93. Sperling H, Debruyne F, Boermans A, Beneke M, Ulbrich E, Ewald S. The POTENT I randomized trial: Efficacy and safety of an orodispersible vardenafil formulation for the treatment of erectile dysfunction. J Sex Med;**7**(4):1497-507

94. Ritchie CW, Bush AI, Mackinnon A, et al. Metal-Protein Attenuation with Iodochlorhydroxyquin (Clioquinol) Targeting Abeta Amyloid Deposition and Toxicity in Alzheimer Disease: A Pilot Phase 2 Clinical Trial. Arch Neurol;**60**(12):1685-91

95. Oertel W, Eggert K, Pahwa R, et al. Randomized, placebo-controlled trial of ADS-5102 (amantadine) extended-release capsules for levodopa-induced dyskinesia in Parkinson's disease (EASE LID 3). Mov Disord;**32**(12):1701-09

96. Olanow CW, Factor SA, Espay AJ, et al. Apomorphine sublingual film for off episodes in Parkinson's disease: a randomised, double-blind, placebo-controlled phase 3 study. 2020;**1**(2):135-44
