# Supplementary material for: Drug-induced orthostatic hypotension: A systematic review and meta-analysis of randomised controlled trials
Source: PLoS Med. 2021 Nov 9;18(11):e1003821. doi: 10.1371/journal.pmed.1003821 (PMC8577726; doi:10.1371/journal.pmed.1003821)
Supplement: S2 Table — Table A. GRADE assessments. Table B. GRADE assessment criteria. (DOCX) [file pmed.1003821.s003.docx]

**Supplementary Data**

**S4 Table A: GRADE assessments**

*Table 3 GRADE assessments*

| Quality assessment | | | | | | | No of patients | | Effect | | Quality | Recommendation |
| --- | --- | --- | --- | --- | --- | --- | --- | --- | --- | --- | --- | --- |
| No of studies | Design | Risk of bias | Inconsistency | Indirectness | Imprecision | Other considerations | Drug | Placebo | 95% CI | Odds ratio |  |  |
| **CCBs association with OH** | | | | | | | | | | | |  |
| 5 | RCT | Most information is from studies at moderate ROB | no | yes (populations) | wide CI | upgraded for confounders subgroup analysis | 372 | 349 | (0.49-1.65) | 0.89 | low | *no increased risk of OH compared to placebo; needs more conclusive evidence (exploring dose)* |
| **ACE/ARBs** | | | | | | | | | | | |  |
| 8 | RCT | Most information is from studies at low ROB | no | yes (populations) | wide CI | upgraded for confounders subgroup analysis | 716 | 532 | (0.54,2.74) | 1.22 | moderate | *no increased risk of OH compared to placebo; needs more evidence (exploring dose, older patients).* |
| **SSRIs and association with OH** | | | | | | | | | | | |  |
| 6 | RCT | Most information is from studies at moderate ROB | no | yes (populations, age) | wide CI | upgraded for confounders subgroup analysis | 1443 | 890 | (0.70,1.45) | 1 | low | *no increased risk of OH compared to placebo; needs more evidence (exploring dose, older patients/T2DM).* |
|  |  |  |  |  |  |  |  |  |  |  |  |  |
| **SGLT-2 inhibitors association with OH** | | | | | | | | | | | |  |
| 10 | RCT | Most information is from studies at moderate ROB | no | no | yes | upgraded for confounders subgroup analysis | 4545 | 3961 | (1.07-1.43) | 1.25 | moderate | *increased risk of OH compared to placebo; needs more conclusive evidence - recommend monitoring for OH in T2DM* |
| **Alpha-adreno receptor blockers association with OH** | | | | | | | | | | | |  |
| 17 | RCT | Most information is from studies at low ROB | yes | no | no | upgraded for confounders subgroup analysis | 4107 | 2569 | (0.78-1.79) | 1.18 | high | *2-fold increased risk of OH compared to placebo, likely greater risk in older population - recommend lower doses of drug for BPH/LUTS in older men & preferably uroselective alpha blockers; regular monitoring* |
|  | **Centrally acting alpha agonists** | | | | | | | | | | |  |
|  |  |  |  |  |  |  |  |  |  |  |  |  |
| 3 | RCT | Most information is from studies at moderate ROB | no | yes (populations; dose) | no | downgraded for smaller studies and one very large sponsored study - at high risk of publication bias; upgraded for large effect size | 1007 | 1013 | (1.55,3.74) | 2.4 | low | *2-fold increased risk of OH compared to placebo; needs more conclusive evidence in community setting; rarely used in practice for hypertension* |
| **Second generation antipsychotics association with OH** | | | | | | | | | | | |  |
| 9 | RCT | Most information is from studies at low ROB | no | yes (populations) | no | upgraded for large effect size | 1663 | 845 | (0.69-2.84) | 1.4 | high | *2-fold increased risk of OH compared to placebo; recommend monitoring for patients at higher risk of OH* |
|  |  |  |  |  |  |  |  |  |  |  |  |  |
| **Beta-blockers** | | | | | | | | | | | |  |
| 8 | RCT | Most information is from studies at moderate ROB | no | yes (population) | no | upgraded for large effect size | 883 | 214 | (2.51, 24.03) | 7.76 | moderate | *significantly increased risk of OH compared to placebo; caution in all patients especially those at higher risk of OH - recommend monitoring* |
| **TCAs association with OH** | | | | | | | | | | | |  |
| 3 | RCT | Most information is from studies at low ROB | no | yes (population age; dose) | no | upgraded for large effect, downgraded for likely publication bias (all small studies) | 136 | 125 | (2.86,13.91) | 6.3 | moderate | *significantly increased risk of OH with medium-high dose TCAs for depression in adults* |

**S5 Table B: GRADE assessment criteria**

| Design | Risk of bias | Inconsistency | Indirectness | Imprecision | Other considerations |
| --- | --- | --- | --- | --- | --- |
| RCT - high | Mostly studies at low ROB  Mostly studies at moderate ROB  Mostly studies at high ROB | Downgrade if the results are not very consistent (test for heterogeneity P>0.1, I^2^ less than 30%) | Downgrade for differences in population/drug/outcome | If the 95% CI excludes a relative risk (RR) of 1.0, precision is adequate. If the 95% CI includes appreciable benefit or harm (an RR of under 0.75 or over 1.25) rating down for imprecision may be appropriate | Rating is modified upward:  - Large magnitude of effect  - Dose response  - Confounders likely minimize the effect Rating modified downward: - Publication bias likely |
