# Supplementary figures and images for: Drug-induced orthostatic hypotension: A systematic review and meta-analysis of randomised controlled trials
Source: PLoS Med. 2021 Nov 9;18(11):e1003821. doi: 10.1371/journal.pmed.1003821 (PMC8577726; doi:10.1371/journal.pmed.1003821)

**Supplementary Data**

**S3 Fig: Risk of bias results**


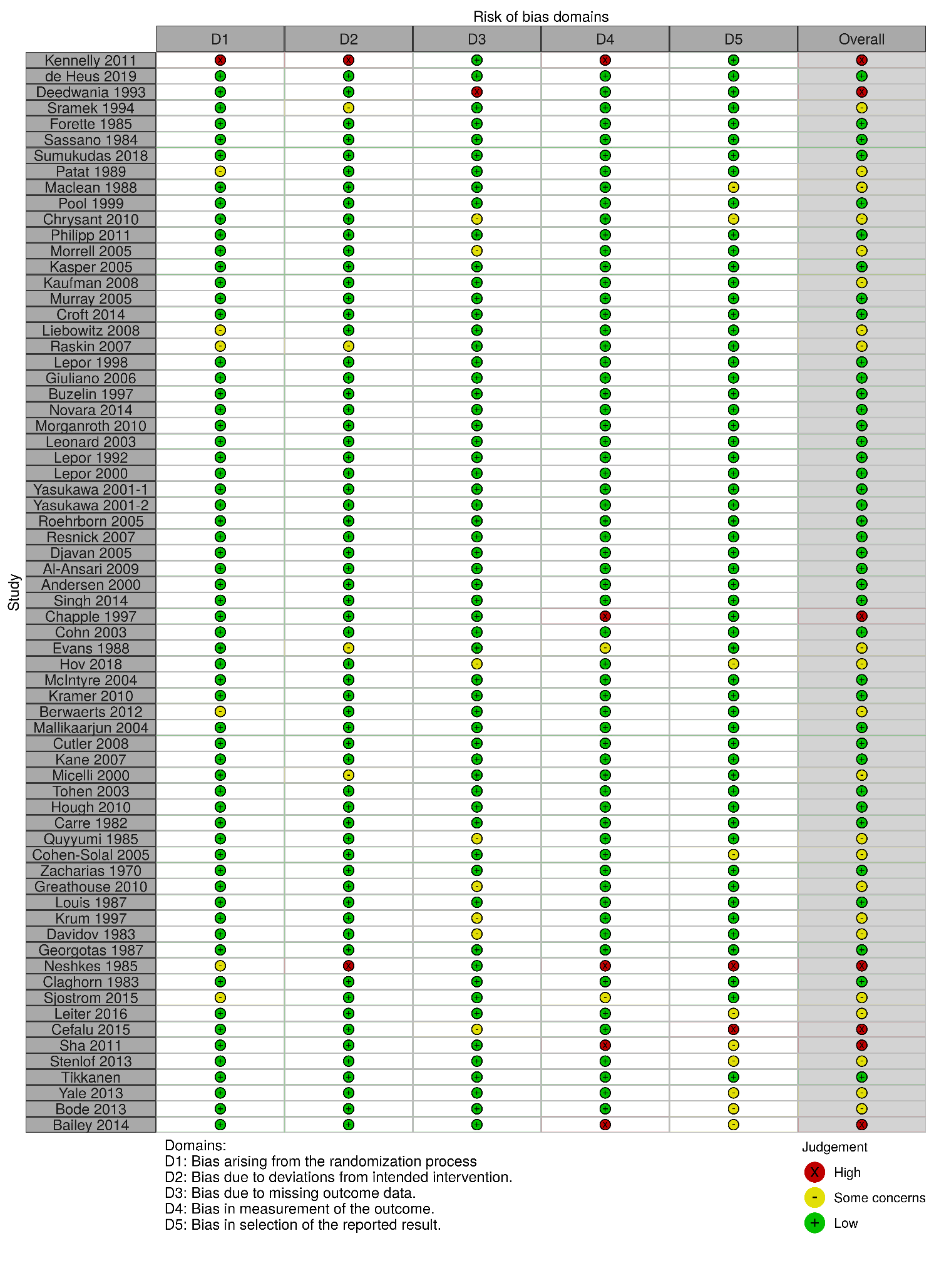

Supplement: S1 Fig — (DOCX) [file pmed.1003821.s004.docx]
