# Supplementary material for: Drug-induced orthostatic hypotension: A systematic review and meta-analysis of randomised controlled trials
Source: PLoS Med. 2021 Nov 9;18(11):e1003821. doi: 10.1371/journal.pmed.1003821 (PMC8577726; doi:10.1371/journal.pmed.1003821)
Supplement: S2 Fig — (DOCX) [file pmed.1003821.s005.docx]

**Supplementary Data**

**S4 Fig: Subgroup analyses and funnel plots**

***Calcium-channel blockers***

*a) trials reporting “measured and validated” OH outcomes only*

*
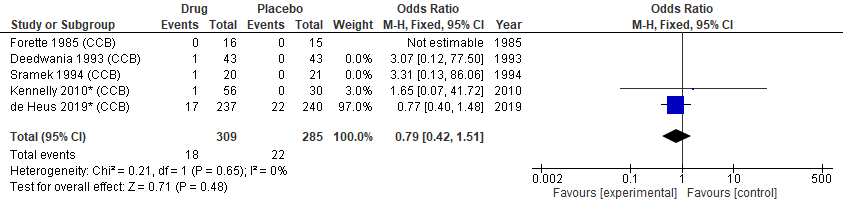
*

*b) trials at low risk of bias*

*
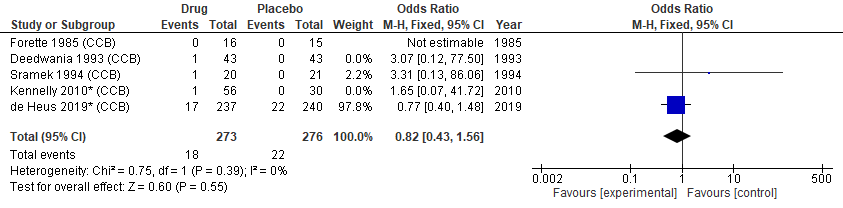
*

*c) older participants ≥ 65 years*

*
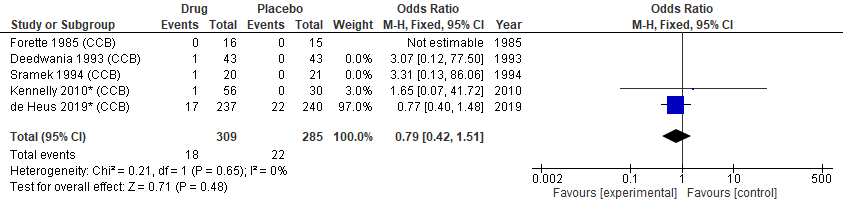
*

*d) participants with cardiovascular conditions*

***
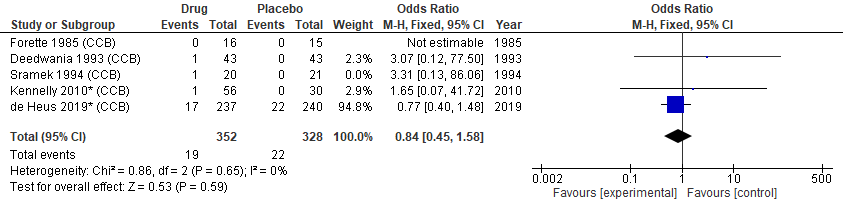
***

***
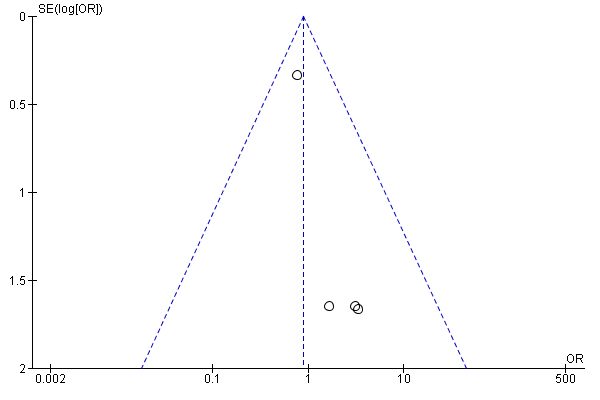
***

***ACE inhibitors/ARBs***

*a) trials reporting “measured and validated” OH outcomes only*


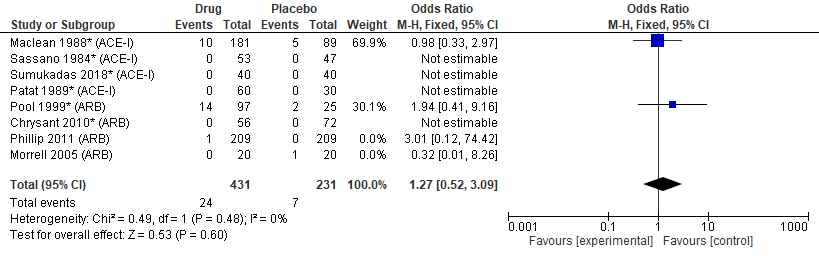


*b) dose*

*low dose*

*
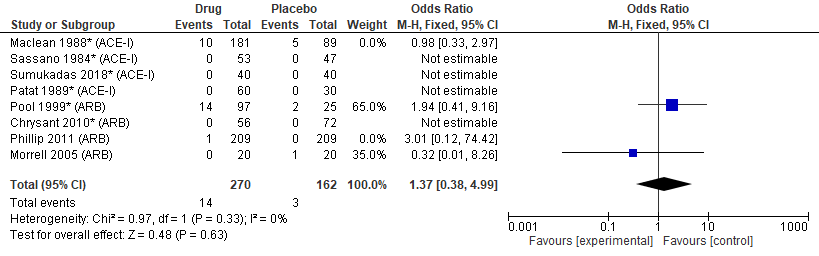
*

*high dose*

*
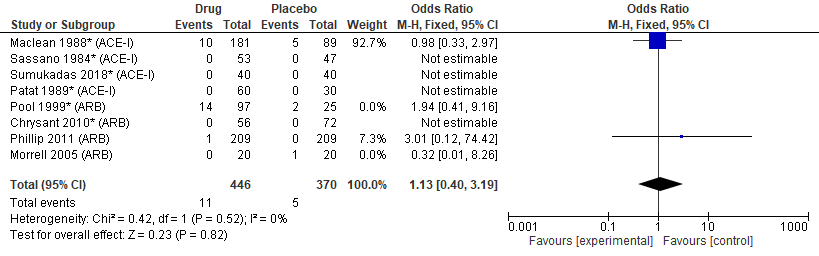
*

*c) populations at greater risk of OH (excluding healthy volunteers)*

*
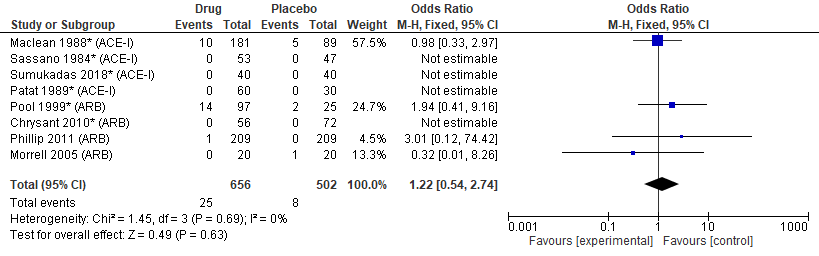
*

*Funnel plot*

*
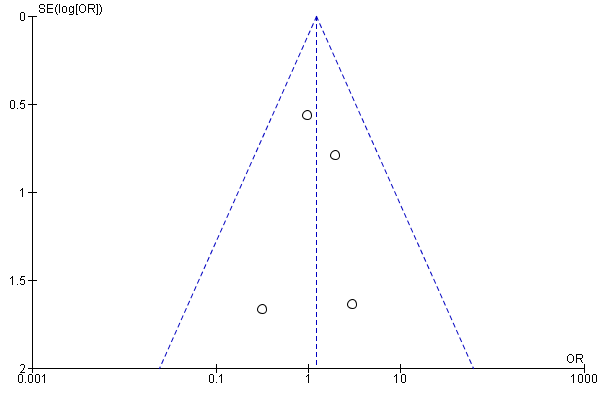
*

***SSRIs/serotonin modulators***

*a) trials reporting “measured and validated” OH outcomes only*

*
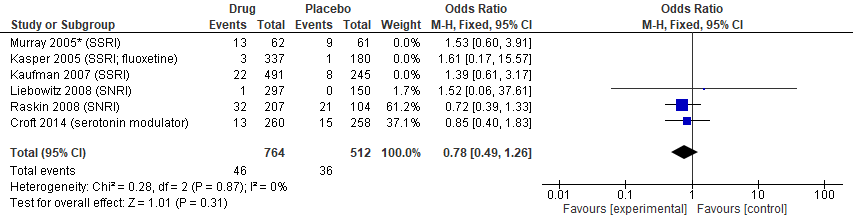
*

*b) drug dose*

*Low dose*

*
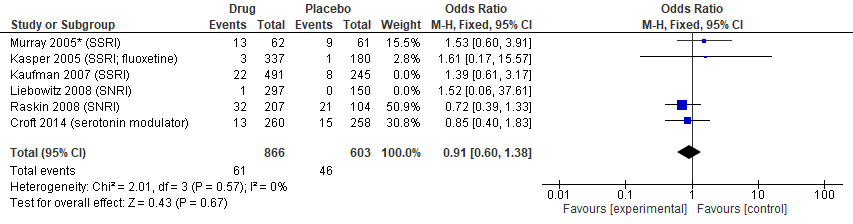
*

*High dose*

*
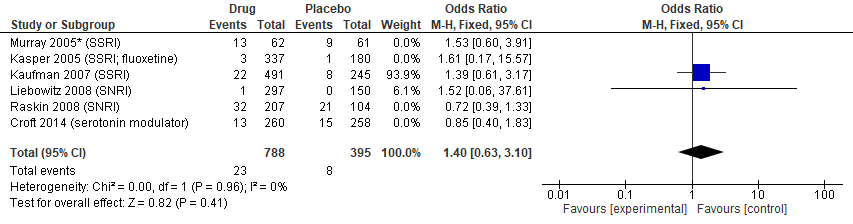
*

*3) older patients ≥ 65 years*

*
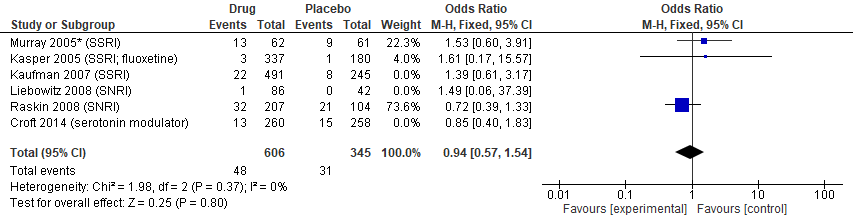
*

*Funnel plot*

*
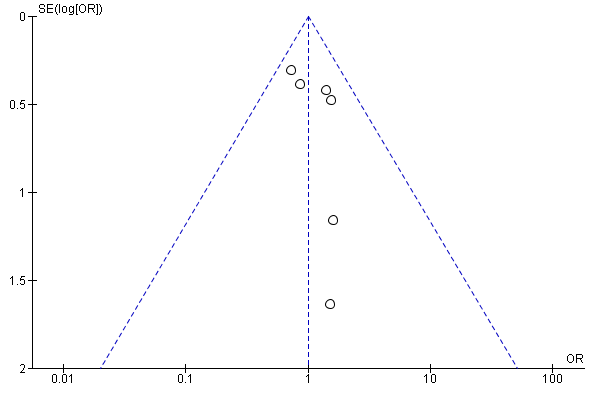
*

***SGLT-2 inhibitors***

*a) trials at low risk of bias (excluding trials with an overall score of “high”).*

*
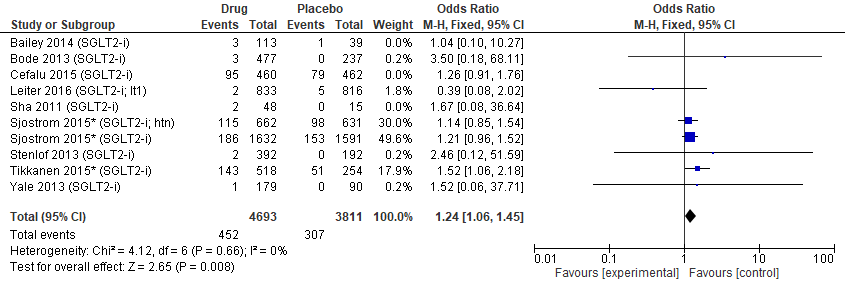
*

*b) trials in patients with T2DM*

*
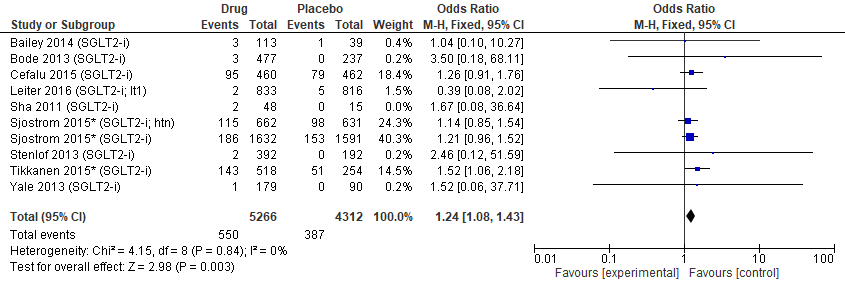
*

*c) trials in patients with T2DM and cardiovascular disease*

*
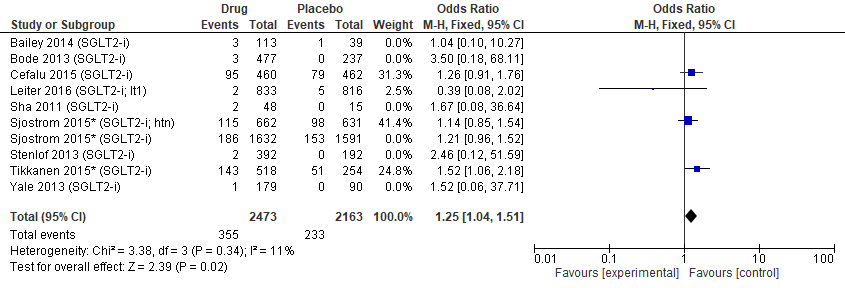
*

*Funnel plot*

*
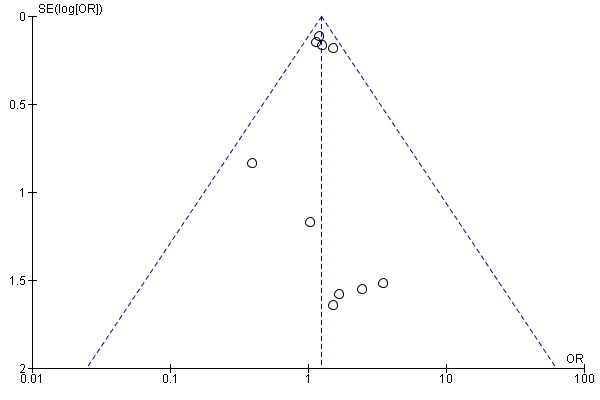
*

***Alpha-adreno receptor blockers***

*1) trials reporting “measured and validated” OH outcomes only*


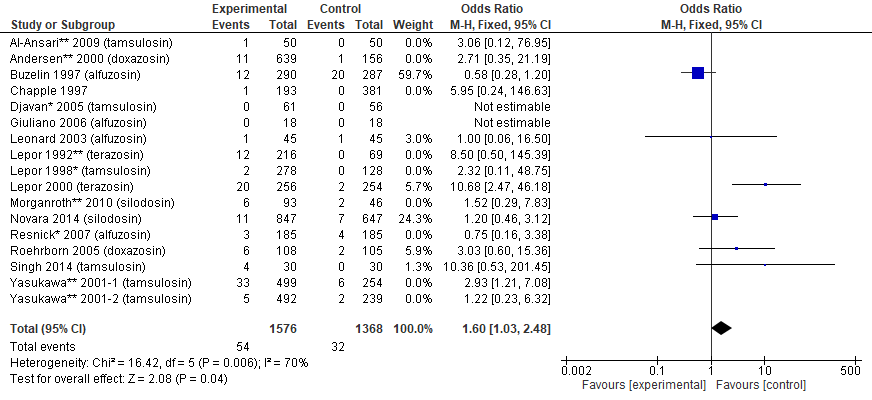


*Funnel plot*

*
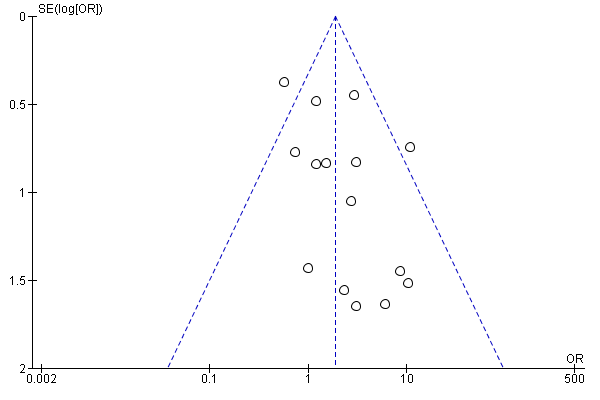
*

***Centrally acting antihypertensives (alpha-agonists)***

*
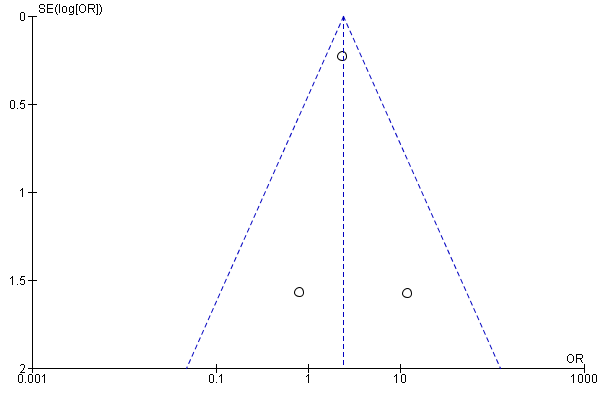
*

***Second generation antipsychotics***

*1) trials reporting “measured and validated” OH outcomes only*

***
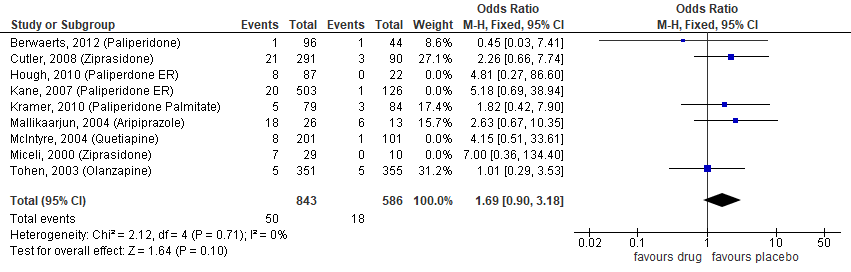
***

*b) dose*

*low dose*

*
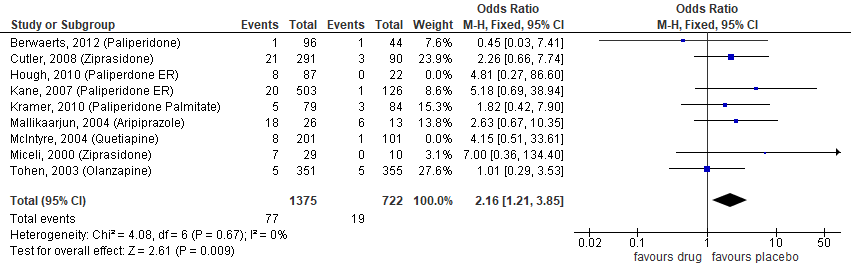
*

*high dose*

*
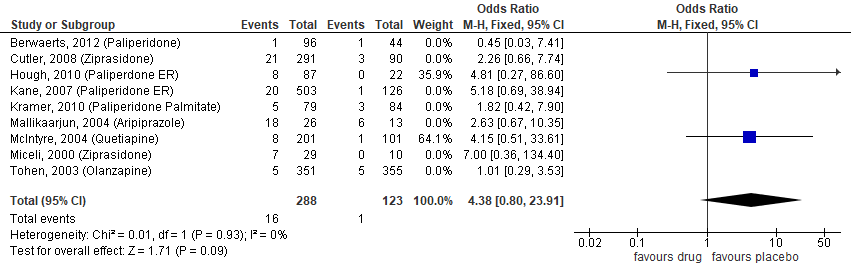
*

*c) populations with mental health conditions (excluding healthy volunteers)*

*
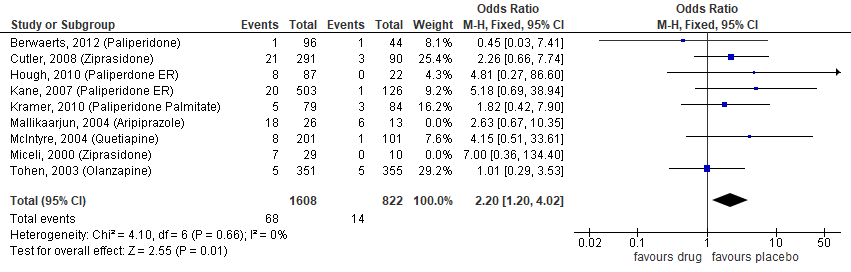
*

*Funnel plot*

*
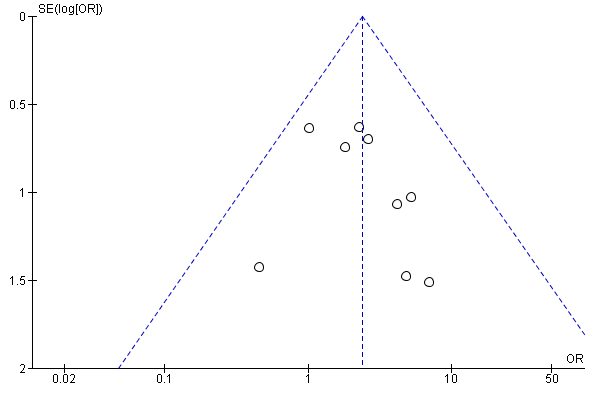
*

***Beta-blockers***

*a) trials with “measured and validated” OH outcomes*

*
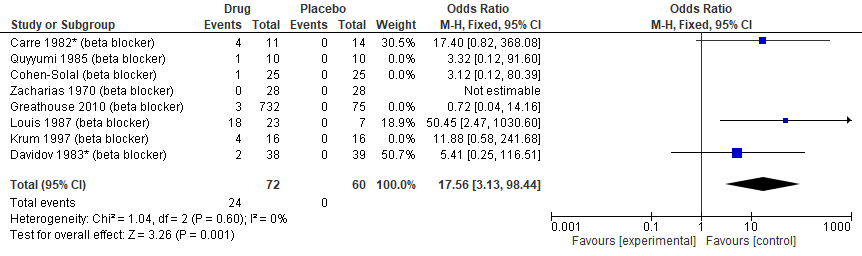
*

*b) low dose (excluding 1 trial with high dose)*

*
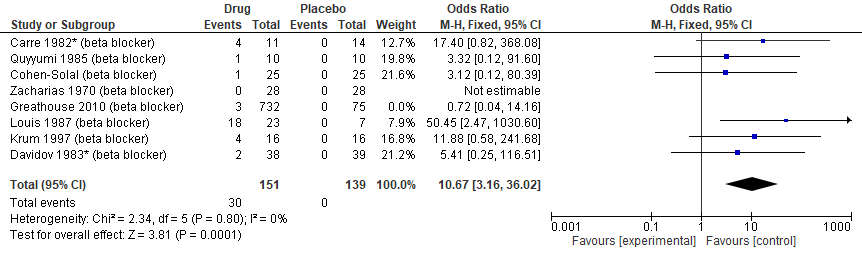
*

*c) population at higher risk of OH (hypertension; angina; heart failure)*

*
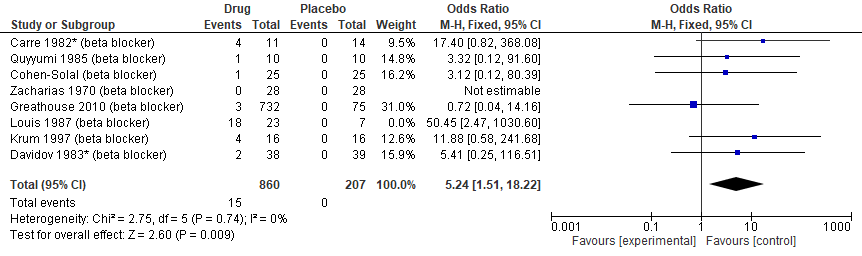
*

*Funnel plot*

*
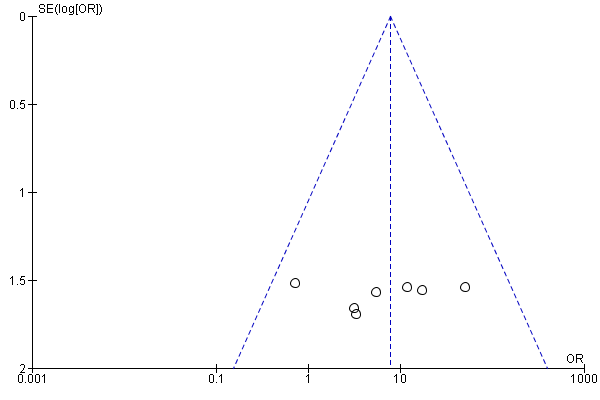
*

*Tricyclic antidepressants (TCAs)*

*Funnel plot*

*
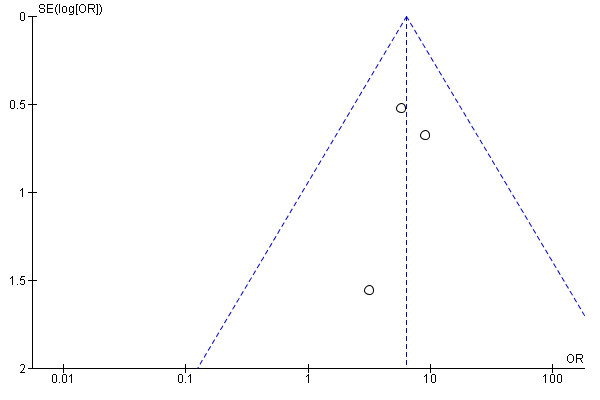
*
